# RNA-Seq Reveals Sex Differences in Gene Expression during Peripheral Neuropathic Inflammation and in Pain Relief from a COX-2 Inhibiting Theranostic Nanoemulsion

**DOI:** 10.3390/ijms24119163

**Published:** 2023-05-23

**Authors:** Brooke Deal, Katherine Phillips, Caitlin Crelli, Jelena M. Janjic, John A. Pollock

**Affiliations:** 1 Department of Biological Sciences, School of Science & Engineering, Duquesne University, Pittsburgh, PA 15282, USA; dealbrooke11@gmail.com (B.D.);; 2 Chronic Pain Research Consortium, Duquesne University, Pittsburgh, PA 15282, USA; 3 Graduate School of Pharmaceutical Sciences, Duquesne University, Pittsburgh, PA 15282, USA

**Keywords:** sex differences, COX-2 inhibitor, celecoxib, RNA-seq, ATF3, neuroinflammation, nanotheranostic, nanoemulsion, transcriptome, rat CCI

## Abstract

Given decades of neuroinflammatory pain research focused only on males, there is an urgent need to better understand neuroinflammatory pain in females. This, paired with the fact that currently there is no long-term effective treatment for neuropathic pain furthers the need to evaluate how neuropathic pain develops in both sexes and how it can be relieved. Here we show that chronic constriction injury of the sciatic nerve caused comparable levels of mechanical allodynia in both sexes. Using a COX-2 inhibiting theranostic nanoemulsion with increased drug loading, both sexes achieved similar reduction in mechanical hypersensitivity. Given that both sexes have improved pain behavior, we specifically explored differential gene expression between sexes in the dorsal root ganglia (DRG) during pain and relief. Total RNA from the DRG revealed a sexually dimorphic expression for injury and relief caused by COX-2 inhibition. Of note, both males and females experience increased expression of *activating transcription factor 3* (*Atf3*), however, only the female DRG shows decreased expression following drug treatment. Alternatively, *S100A8* and *S100A9* expression appear to play a sex specific role in relief in males. The sex differences in RNA expression reveal that comparable behavior does not necessitate the same gene expression.

## 1. Introduction

Until recently, the vast majority of preclinical pain studies were performed exclusively in male rodent models with fundamental neurobiological studies exhibiting one of the biggest sex disparities [1]. Even though, in humans, females are more often affected by neuropathic pain [2,3], the years of disproportionate research has led the chronic pain field to develop a male-biased body of research [4]. For example, a recent review of pain literature found that for nearly a decade, the research published in a particular journal only reported results from male rodents or did not report sex as a variable 82% of the time [4]. In fact, this review summarized that for 127 different pain studies, an overwhelming 72.4% found their hypotheses to be true in males but not in females [4]. 

This biased body of research has skewed the understanding of pain biology and, in turn, led to issues in the translational aspect of therapeutics [5,6]. Currently, medications used to treat pain include anticonvulsants, antidepressants, non-steroidal anti-inflammatory drugs (NSAIDs), and opioids [7,8]. However, for each, their adverse side effects often limit long-term use [9], and a growing body of research reveals sex differences in drug action [10,11]. In order to help develop safer and more effective therapeutics, sex differences should be closely evaluated examining the underlying biology of neuropathic hypersensitivity (defined as pain-like behavior) and of the biological consequences of its relief.

With the National Institute of Health’s requirement for investigators to include sex as a biological variable (NOT-OD-15-102), more sex differences have been revealed in the development of neuropathic inflammatory hypersensitivity and pain [12,13]. A few studies have evaluated the expression differences between sexes experiencing neuroinflammatory pain [14,15,16,17,18,19]. For example, during neuroinflammation, RNA expression patterns suggest an involvement of T cells, as well as activation of immune signaling pathways, changes in neuronal plasticity, and enrichment from other gene pathways involved in the neuronal immune crosstalk [15,18,20]. However, the sex-specific differential expression of genes responding to pain relief therapy between sexes has yet to be evaluated. 

Considering that COX-2 inhibiting NSAIDs are one of the most commonly prescribed classes of pain-relieving drugs [21], and that over 80% of US adults reported using OTC pain medications each year [7], careful examination of the biological differences in pain relief between males and females is warranted. Previously, it has been hypothesized that NSAIDs and COX-2 inhibitors were ineffective as treatment for neuropathic pain [22], nonetheless, our studies have repeatedly shown that a novel COX-2 inhibiting theranostic nanoemulsion containing celecoxib (CXB-NE) is able to relieve mechanical allodynia caused by neuroinflammation at low, non-toxic dose in males by delivering celecoxib directly to macrophages [11,23,24,25,26,27]. In our previous work using chronic constriction injury (CCI) of the rat sciatic nerve, we found that both males and females exhibited similar levels of hypersensitivity after injury as well as both sexes obtaining significant relief with a single dose of 0.24 mg/kg of celecoxib as 1X CXB-NE 11]. However, the amount of relief is different between sexes, with males experiencing about 5 days of relief and females achieve only partial relief for a shorter duration [11]. 

Here we report that by employing a modified nanoemulsion (NE) with increased drug loading of 10 times over previously reported [11,23,24,25,26,27]. With males and females achieving similar relief from hypersensitivity, differences in gene expression will reveal the sex differences in the underlying biology of pain and pain relief without interference from differences in the degree of pain relief. Thus, the focus of this current study is on sex differences of the underlying biology as revealed by the transcriptional differences in the dorsal root ganglia (DRG), for male and female rats exhibiting CCI hypersensitivity compared to animals experiencing relief. 

The total RNA for each animal’s affected L4/L5 DRG revealed significant differential expression in transcriptomes of each condition when compared to controls. Here we report on several hundred genes that exhibit differential expression in CCI hypersensitive females as compared to naïve females, distinct from that of CCI hypersensitive males compared to naïve males. These genes represent those that are responsive to neuroinflammation. Furthermore, we find that gene expression that is responsive to celecoxib theranostic nanoemulsion (10X CXB-NE ), which provides relief from hypersensitivity from neuronal injury, also differs between sexes. These differentially expressed genes we refer to as relief induced expression changes. For example, the cAMP response element-binding (CREB) protein, *activating transcription factor 3* (*Atf3*), is significantly increased in the hypersensitive state for both males and females. However, when COX-2 inhibiting theranostic nanoemulsion is present, *Atf3* mRNA and protein are significantly reduced in females, but not so in males. These observations demonstrate fundamental differences in the gene expression associated with pain-like behaviors and in differential gene expression that is evident between sexes associated with NSAID-induced relief. Our data also illustrates that even with these sex-specific differences in gene expression, this dose of the celecoxib theranostic nanoemulsion is sufficient to reduce the number of infiltrating macrophages at the site of injury in both males and females in a manner that correlates with the behavioral relief from hypersensitivity that is evident.

## 2. Results

### 2.1. 10X CXB-NE Treatment Alleviates Mechanical Hypersensitivity Caused by Neuroinflammation in Both Males and Females and Reduces the Infiltration of Macrophages at the Site of Injury

**Figure 1:**
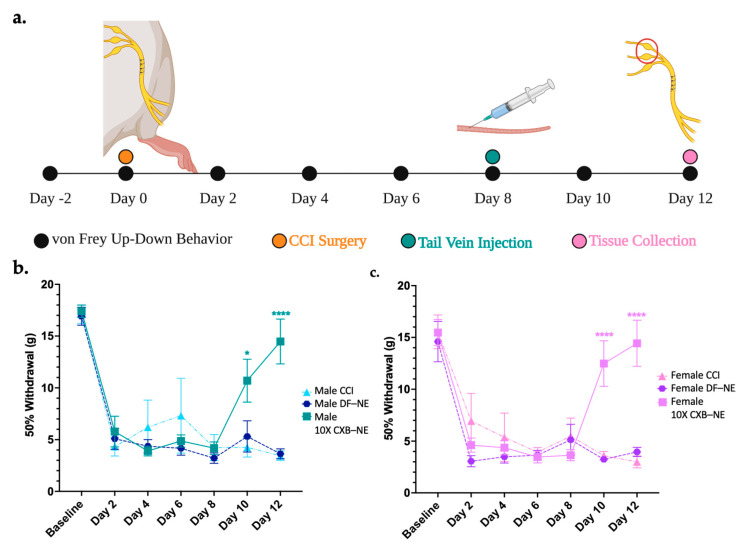
Mechanical allodynia relieved by single dose of Celecoxib theranostic nanoemulsion in both sexes. (**a**) Timeline of animal procedures, dosage treatment, and tissue collection for male and female cohorts. Estrus cycle tracked on days 8–12 and the females were found to be asynchronous with no apparent effect on behavior. (**b**) Male mechanical allodynia observed post-surgery becomes hypersensitive by day 2 and is significantly relieved following 10X CXB-NE on day 8 when compared to CCI and DF-NE groups. (Number of animals included are CCI *n* = 4, DF-NE *n* = 7, and 10X CXB-NE *n* = 7). (**c**) Female von Frey behavioral data shows post-surgery hypersensitivity occurring by day 2 aligning with the same trajectory and the same degree of hypersensitivity as the males. Following theranostic nanoemulsion treatment on day 8, significant relief from mechanical allodynia is evident when compared to CCI and DF-NE groups (Number of animals included are CCI *n* = 6, DF-NE *n* = 7, and 10X CXB-NE *n* = 7). Data displayed as averaged ± SEM. * ≤0.05, **** ≤0.0001. Male and female behavioral data was compared using a two-way ANOVA with a Tukey’s post hoc analysis. (**a**) created with BioRender.com (accessed on 6 October 2022), and (**b**,**c**) created using Prism v9.4.1.

The von Frey up-down analysis was used to assess the mechanical allodynia induced by CCI of the right sciatic nerve and to demonstrate the relief achieved by 10X CXB-NE treatment. The animal procedure timeline is illustrated in Figure 1a. Briefly, baseline behavior was acquired 2 days prior to surgery and on day 0, preceding the CCI surgery. These two days were averaged to give a baseline 50% withdrawal threshold for each animal, which was 16–17 g for both sexes (Figure 1b,c). Following CCI, all groups that underwent the surgery showed decreased 50% withdrawal thresholds that levels off at about 5 g in both sexes. Similar time courses in the decrease in withdrawal were seen between both sexes (2-way ANOVA with Tukey post hoc, *p* > 0.9999 day 6, *p* > 0.9999 day 8). (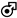 DF-NE day 6 = 4.17 g, STD = 1.76, 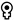 DF-NE day 6 = 3.64, STD = 1.24, 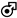 DF-NE day 8 = 3.22 g, STD = 1.33, 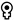 DF-NE day 8 = 5.13, STD = 3.94). This indicates that both males and females are experiencing the same degree of provoked hypersensitivity resulting from the CCI surgery. Tail vein injections of either 10X CXB-NE or drug free nanoemulsion (DF-NE) were delivered on day 8 following von Frey behavior analysis. Receiving DF-NE did not decrease mechanical withdrawal threshold, nor did it produce any type of relief. On days 10 and 12, males (Figure 1b) and females (Figure 1c) both experienced significant relief when compared to their CCI counterparts (Day 10 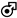 *p* = 0.0338, Day 12 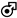 *p* < 0.0001, Day 10 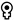 *p* < 0.0001, Day 12 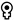 *p* < 0.0001) and DF-NE counterparts (Day 10 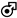 *p* = 0.0404, Day 12 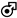 *p* < 0.0001, Day 10 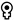 *p* < 0.0001, Day 12 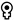 *p* < 0.0001). We also find that on day 12, the 10X CXB-NE treated males and females exhibit no statistically significant difference in behavior as compared to their naïve baseline (2-way ANOVA with Tukey post hoc analysis, *p* > 0.9999). (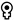 10X CXB-NE day 12 = 14.43 g, STD = 5.88, 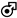 = 14.5 g, STD = 5.73). Furthermore, by assessing the female estrus cycle, we find that in this case, all the females were asynchronous, and their estrus cycle status did not correlate to any bias of their behavior (Supplementary Figure S1). Similar to what we have previously reported [11], the individual’s estrus cycle does not appear have an overt effect on the hypersensitivity behavior or the female response to CXB-NE. Dorsal root ganglia and sciatic nerves were recovered for subsequent analysis on day 12 because it is the day of maximum relief. 

**Figure 2:**
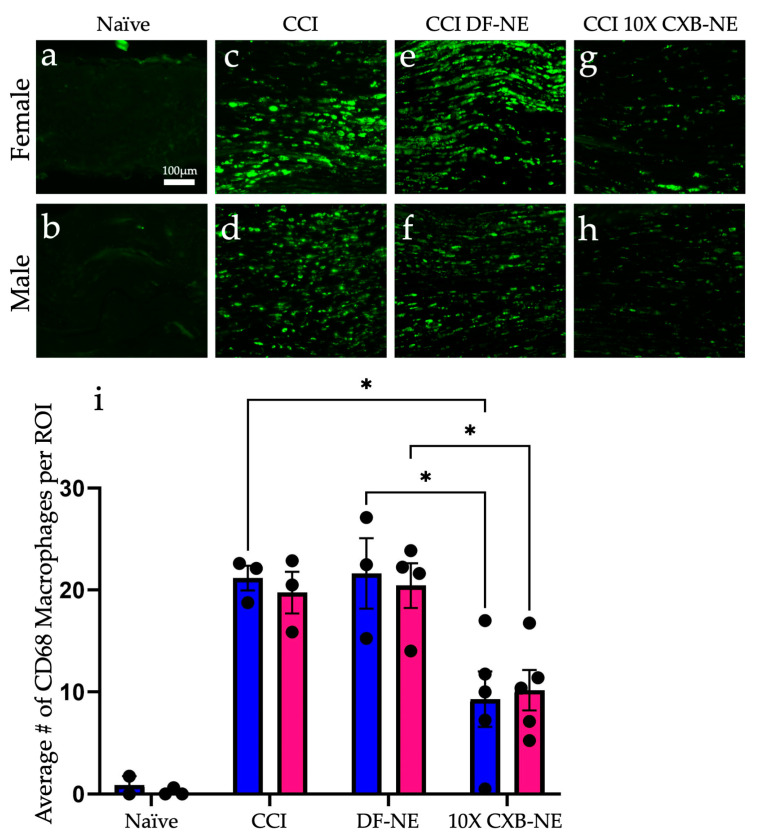
Celecoxib theranostic nanoemulsion (10X CXB-NE) reduces the infiltration of CD68 positive macrophages in the injured sciatic nerve equally for both sexes. (**a**) The non-surgical naïve female sciatic nerve exhibits few if any CD68 positive macrophages. (**b**) The non-surgical naïve male sciatic nerve typically exhibits few CD68 positive macrophages. (**c**) The female experiencing CCI exhibits a significant number of infiltrating CD68 positive macrophages in the injured nerve. (**d**) The CCI male exhibits a significant number of infiltrating CD68 positive macrophages in the injured nerve. (**e**) The female CCI sciatic nerve treated with drug-free nanoemulsion (DF-NE) exhibits a significant number of CD68 cells within the fasciculated nerve. (**f**) The male CCI sciatic nerve similarly treated with DF-NE exhibits numerous CD68 positive cells within the fasciculated nerve. (**g**) The female CCI sciatic nerve treated with 10X CXB-NE exhibits a significant reduction in the number of CD68 cells as compared to its CCI and DF-NE counterparts. (**h**) The male CCI sciatic nerve treated with 10X CXB-NE exhibits a reduced number of CD68 positive cells within the nerve as compared with its CCI and DF-NE counterparts. (**i**) For each animal, the number of CD68 positive cells were counted in 8 regions of interest (ROI) of equal size (140 µm × 140 µm). The average number of cells per ROI for each animal is plotted as a dot. (Naive: Male *n* = 2, Female *n* = 3. CCI: Male *n* = 3, Female *n* = 3. DFNE: Male *n* = 3, Female *n* = 4. CXBNE: Male *n* = 4, Female *n* = 5). In order to assess treatment effect per sex, a one-way ANOVA was performed with a Tukey’s post-hoc analysis. Data is displayed as averages ±SEM. * ≤0.05. Bar = 100 µm.

Immunohistochemical assessment on day 12 of CD68 positive macrophages at the site of injury revealed a similar reduction in the prevalence of these infiltrating immune cells in both male and female CCI sciatic nerves when the COX-2-inhibiting theranostic nanoemulsion was present (Figure 2). Non-surgical naïve animals exhibit few, if any CD68 positive macrophages within the fasciculated nerve bundle of the sciatic nerve (Figure 2a,b). When experiencing CCI, both males and females exhibit a significant number of infiltrating CD68 positive macrophages in the injured nerve. The comparable degree of inflammation as visualized by these macrophages correlates with the behavior for those animals that are still hypersensitive when treated with the DF-NE in both sexes (Figure 2e,f). When treated with 10X CXB-NE, both males and females exhibit similar degree of relief from hypersensitivity and similar reduction in the number of infiltrating macrophages in the sciatic nerve (Figure 2g,h). Analysis of the number of infiltrating CD68 positive cells per region of interest (Figure 2i) reveals that the naïve males and females have few if any detectable CD68 cells present. However, the relative number of CD68 macrophages is high for both male and female CCI nerves; equal to that what is observed for DF-NE treated CCI nerves. This high degree of inflammation correlates with the hypersensitivity detected in the von Frey assessment of the mechanical allodynia. Yet, when CCI male and female rats receive 10X CXB-NE, which provides relief from hypersensitivity comparable to their pre-surgical baseline, the reduced number of CD68 positive macrophages in the nerves of both sexes is the same (Figure 2i).

### 2.2. Mechanical Hypersensitivity Induced by Neuroinflammation and Relief following 10X CXB-NE Treatment Causes a Shift in Gene Expression in the DRG

The DRG corresponding to the right sciatic nerve were collected on day 12 (Figure 1a) and total RNA was extracted from both the RL4 and RL5 DRG for each animal. The RNA from the RL4 and the RL5 DRG were sequenced together as one sample per animal. Samples were sequenced using the NextSeq High Output flow cell instrument (Illumina Inc., San Diego, CA, USA). An average of 74 million reads per sample were generated with lengths of 75 bp; 85% of the raw reads on average were then mapped to the *Rattus norvegicus* (mRatBN7.2) genome using CLC Genomics Workbench 22.0.2 (Qiagen, Hilden, Germany). 

**Figure 3:**
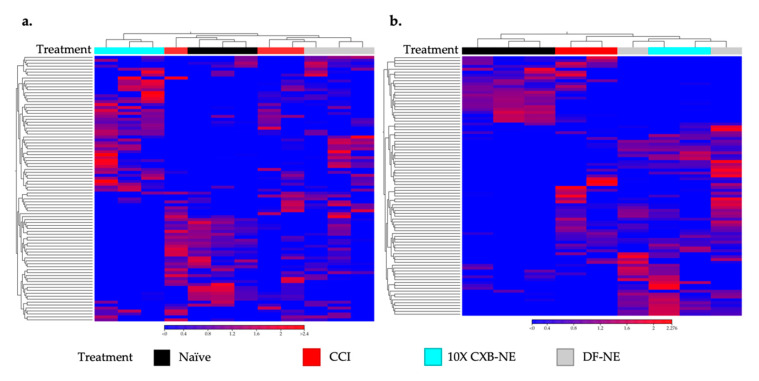
To reveal whether the treatments effect the DRG transcriptomes more in one group or sex than another, heatmaps displaying the top 90 genes with the highest coefficients of variation in male and female DRGs were compared. (**a**) Euclidean linkage was used to display the normalized log counts per million (CPM) values to show the similarities and differences in male individual animals (male naïve *n* = 3, male CCI *n* = 3, male DF-NE *n* = 3, male 10X CXB-NE *n* = 3). (**b**) Female treated individual animals displayed along the *x*-axis separated by their Euclidean distance of their gene’s normalized log CPM values (female naïve *n* = 3, female CCI *n* = 2, female DF-NE *n* = 2, female 10X CXB-NE *n* = 2). Heatmaps made in CLC Genomics Workbench 22.0.2.

In this case, gene expression heatmaps were used to look at the relatedness of the individual transcriptomes from each animal and how the treatment groups were related to one another. This is important to see whether pain or treatment effects transcriptomes more in one group or sex than another. CLC Genomics Workbench was used to make hierarchically clustered heatmaps of both male (Figure 3a) and female (Figure 3b) rat transcriptomes. Using Euclidean distance and complete linkage of clusters, the top 90 genes (genes listed in Supplementary Tables S1 and S2) with the highest coefficient of variation were used to cluster rat DRG transcriptomes by relatedness. As seen in Figure 3a, male CCI and DF-NE cluster closest together sharing the most related transcriptomes while 10X CXB-NE animals are the least related to the other three groups having the most unique gene expression. Figure 3b shows a different pattern in the treated females where CCI is distinct from naïve, and 10X CXB-NE and DF-NE are most closely related. 

**Figure 4:**
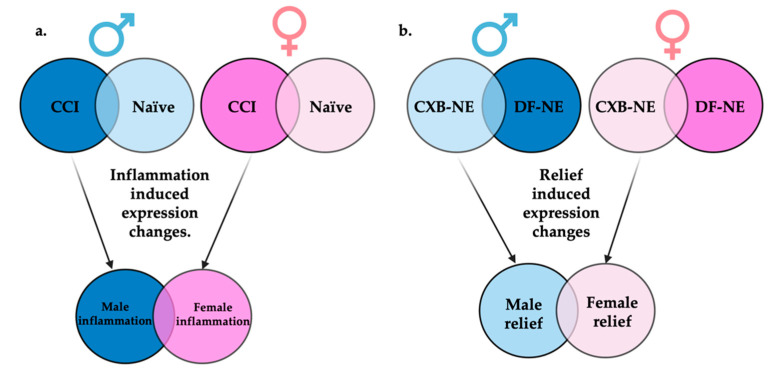
Display of comparisons made between RNA-seq generated gene sets. (**a**) Genes differentially expressed between CCI and naïve of the same sex will be termed inflammatory induced gene expression changes. When these inflammatory gene sets are compared between sexes, genes uniquely differentially expressed in males or females can be identified. (**b**) Genes differentially expressed between 10X CXB-NE and DF-NE of the same sex will be termed relief induced gene expression changes. When these gene sets are compared between sexes, genes uniquely differentially expressed during relief in males or females associated can be identified. Figure made with BioRender.com (accessed on 6 October 2022).

Figure 4 illustrates how differential gene expression analyses were performed to reveal those gene sets that are differentially expressed during male neuroinflammation (CCI vs. naïve), female neuroinflammation (CCI vs. naïve), male neuroinflammatory relief from hypersensitivity (10X CXB-NE vs. DF-NE), and female neuroinflammatory relief from hypersensitivity (10X CXB-NE vs. DF-NE). With the genes filtered by fold change ≥±1 and the adjusted *p*-value (false discovery rate (FDR)) of ≤0.05, there were 265 differentially expressed genes (DEGs) in male neuroinflammation, 977 DEGs in female neuroinflammation, 333 DEGs in male neuroinflammatory relief, and 16 DEGs in female neuroinflammatory relief. These gene sets were then compared between sexes to look at gene expression changes unique to males, gene expression changes unique to females, and shared changes in gene expression across conditions. 

### 2.3. Female Neuroinflammation

CLC Genomics Workbench was used to perform a differential expression analysis between female CCI DRG transcriptomes and female naïve DRG transcriptomes. The genes differentially expressed between the two would be those attributed to neuroinflammation. Whole transcriptomes were compared and normalized using the internal normalization method of trimmed mean of M values (TMM). 

**Figure 5:**
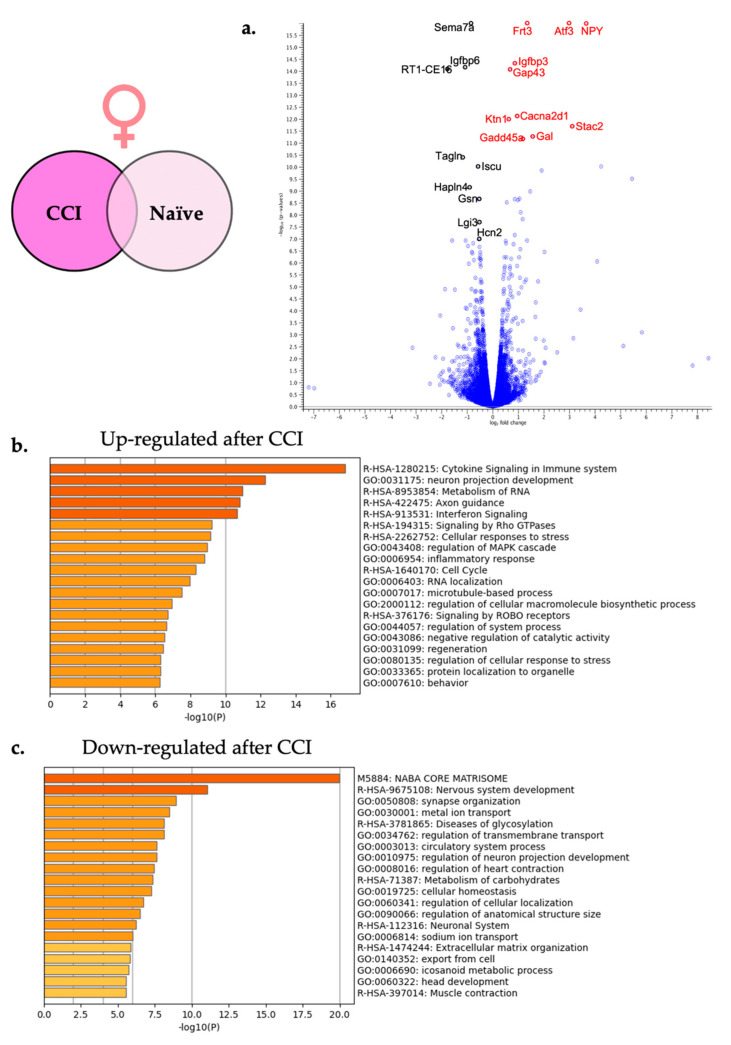
Neuroinflammation in females causes differential gene expression in the affected DRG. (**a**) For visual clarity, the volcano plot features all of the differentially expressed genes due to neuroinflammation in the female DRG and highlights the top 10 significantly up-regulated (red) and top 10 significantly down-regulated genes (black). (**b**) Metascape enrichment analysis of the top 20 up-regulated biological pathways following induction of neuroinflammation. (**c**) Metascape enrichment analysis of the top 20 down-regulated biological pathways following induction of neuroinflammation.

Filtering using the FDR *p*-value ≤ 0.05 and fold change of greater than ±1 revealed that a total of 977 genes were differentially expressed following CCI in the female DRG. A total of 485 genes were significantly up-regulated, and 492 genes were significantly down-regulated. To present some examples, the top 25 significantly up-regulated (Table 1) and down-regulated (Table 2) genes are shown. These DEGs were then displayed using a volcano plot in Figure 5a with the top 10 DEGs highlighted. By focusing on the genes that exhibit differential expression with the greatest FDR significance, we identify some genes with very large fold-change, which represent genes that are either turned on or off, as well as genes with modest fold-change of less than 2 that are nonetheless significant.

In order to better identify which classes of genes or pathways were overrepresented by the differentially expressed genes, a gene set enrichment analysis was performed using Metascape (Metascape.org). Metascape combines multiple enrichment analyses platforms including Gene Ontology (GO) processes, Kyoto Encyclopedia of Genes and Genomics (KEGG) pathways, Reactome gene sets, Cytoscape and NCBI. All 977 significantly differentially expressed genes in females were included in the pathway enrichment analysis with the upregulated genes being evaluated in one analysis and the downregulated genes being evaluated in a second analysis.

The two lists of significantly up-regulated and significantly down-regulated female specific genes were analyzed separately. Figure 5b shows the top 20 enriched pathways that were significantly up-regulated including cytokine signaling in immune system (R-HSA-1280215) and neuron projection development (GO:0031155) among others. Figure 5c shows the top 20 enriched down-regulated pathways including the synapse organization (GO:0050808) and the nervous system development (R-HSA-9675108). 

### 2.4. Male Neuroinflammation

**Figure 6:**
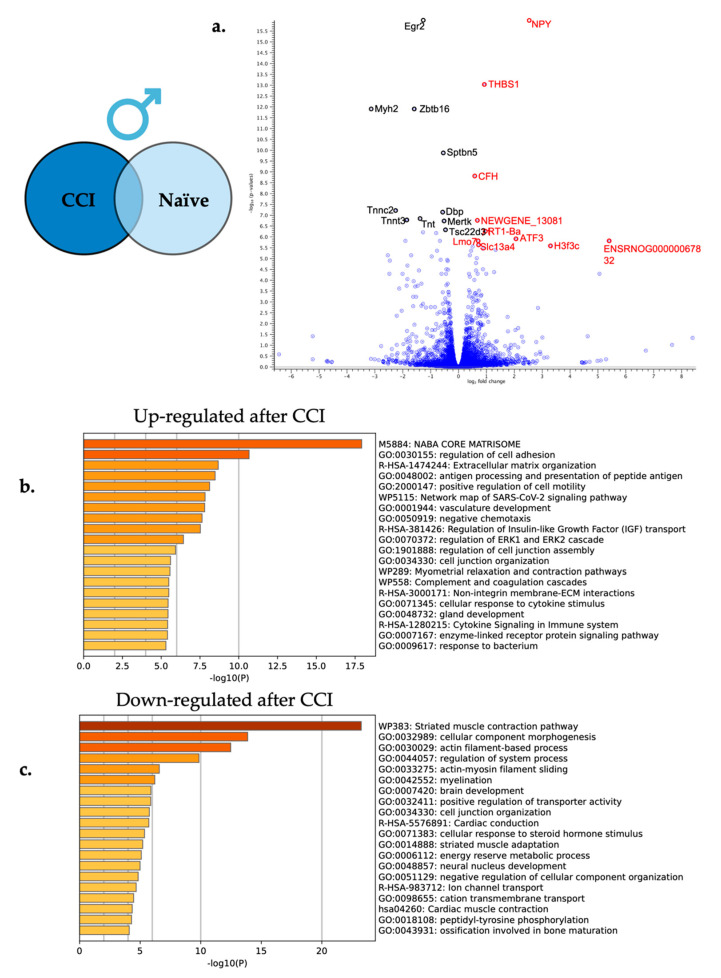
Neuroinflammation in males causes differential gene expression in the affected DRG. (**a**) For visual clarity, the volcano plot features all the differentially expressed genes due to neuroinflammation in the male DRG with a highlight of the top 10 significantly up-regulated (red) and top 10 significantly down-regulated genes (black). (**b**) Metascape enrichment analysis of the top 20 up-regulated biological pathways following induction of neuroinflammation. (**c**) Metascape enrichment analysis of the top 20 down-regulated biological pathways following induction of neuroinflammation.

Using a differentially expressed gene analysis as described above, expressed RNA sequences from male CCI DRG were compared to male naïve DRG to reveal differentially expressed genes altered by neuroinflammation. From this comparison, a total of 265 genes were differentially expressed following peripheral neuroinflammation with 126 genes significantly up-regulated and 139 genes significantly down-regulated. Based on statistical significance, the top 25 up and down-regulated genes are featured in Table 3 and Table 4 with the top 10 significantly up and down-regulated highlighted in the volcano plot in Figure 6a. 

Enriched biological processes were elucidated using Metascape gene set enrichment analysis using express analysis parameters. Again, all 265 significantly differentially expressed genes in males were included in the pathway enrichment analysis with the upregulated genes being evaluated in one analysis and the downregulated genes being evaluated in a second analysis. Overrepresented pathways in both the up and down-regulated differentially expressed gene sets are shown in Figure 6b,c. Some of the enriched up-regulated biological processes include cytokine signaling in the immune system (R-HSA-1280215) and regulation of cell adhesion (GO:0030155), while some down-regulated processes include myelination (GO:0042552) and actin-filament based process (GO:0030029).

### 2.5. Female Neuroinflammatory Relief

**Figure 7:**
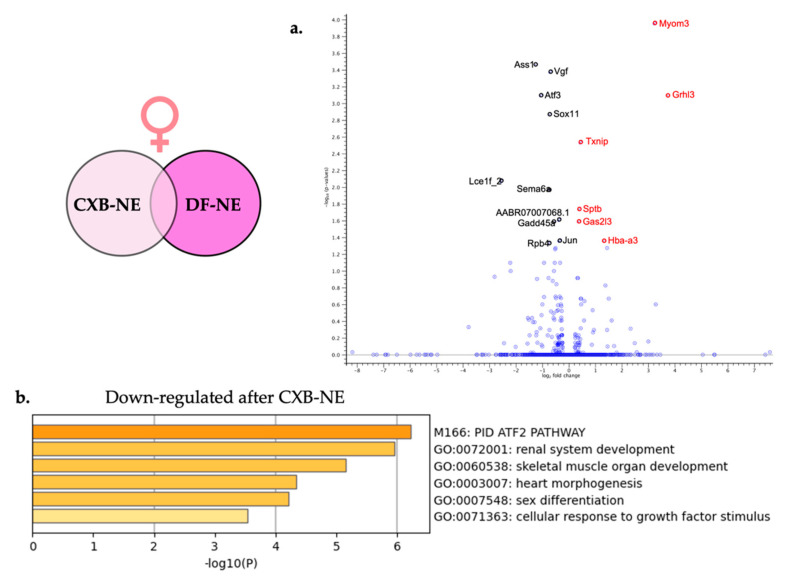
Relief from neuroinflammation from COX-2 inhibiting theranostic nanoemulsion in females causes differential gene expression in the affected DRG. (**a**) For visual clarity, the volcano plot features all of the differentially expressed genes due to relief of neuroinflammation in the female DRG and highlights the top 6 significantly up-regulated (red) and top 10 significantly down-regulated genes (black). (**b**) Metascape enrichment analysis of the down-regulated biological pathways following reduction of neuroinflammation resulting from COX-2 inhibition.

Differential gene expression brought on by relief of peripheral neuroinflammation in females (Figure 1c) was examined using CLC Genomics Workbench to compare the transcriptomes from female 10X CXB-NE DRG against female DF-NE DRG. Using the same filtering parameters that were used in the analysis of neuroinflammation (FDR *p*-value ≤ 0.05 and a fold change greater than ±1), this analysis showed a total of 16 DEGs. Of the 16 DEGs, 6 of them were significantly up-regulated (Table 5) and 10 of them were significantly down-regulated (Table 6). These 16 significant DEGs are featured on the volcano plot in Figure 7a.

Metascape gene enrichment analysis using all 16 significantly differentially expressed genes was performed as described earlier. The two separate lists of up-regulated DEGs and down-regulated DEGs did not show any enriched biological processes for up-regulated genes involved in female neuroinflammatory relief; however, 6 enriched biological processes were revealed from the down-regulated genes. These enriched biological processes include PID ATF-2 pathway (M166) and cellular response to growth factor stimulus (GO:0071363).

### 2.6. Male Neuroinflammatory Relief

**Figure 8:**
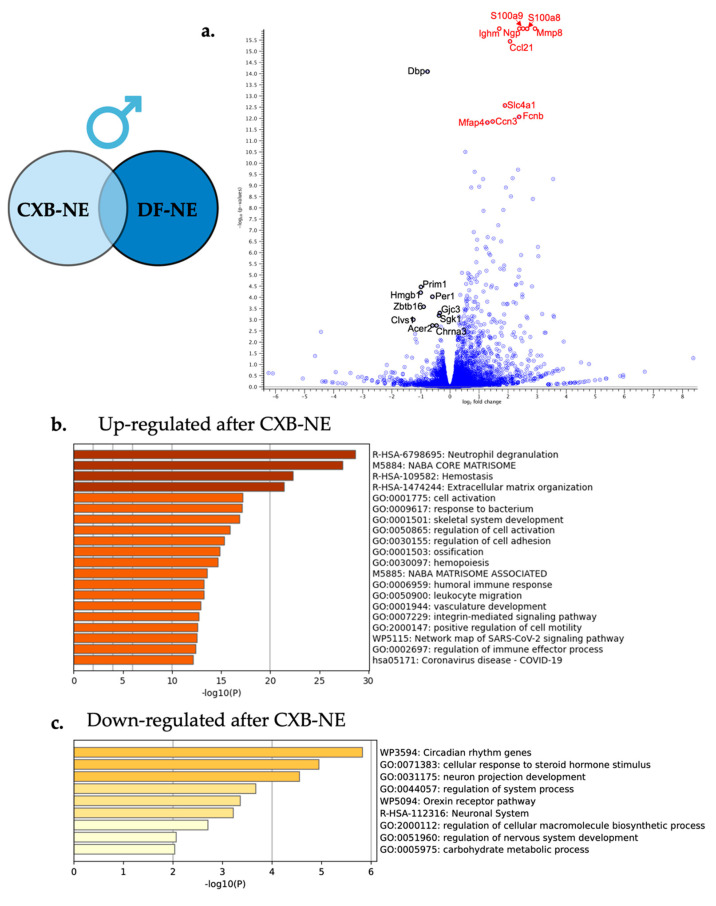
Relief from neuroinflammation by COX-2 inhibiting theranostic nanoemulsion in males causes differential gene expression in the affected DRG. (**a**) For visual clarity, the volcano plot features all the differentially expressed genes due to neuroinflammatory relief in the male DRG with a highlight of the top 10 significantly up-regulated (red) and top 10 significantly down-regulated genes (black). (**b**) Metascape enrichment analysis of the top 20 up-regulated biological pathways following neuroinflammatory reduction via COX-2 inhibition. (**c**) Metascape enrichment analysis of the 9 down-regulated biological pathways following reduction of neuroinflammation resulting from COX-2 inhibition.

Differential gene expression analysis comparing male 10X CXB-NE DRG transcriptomes to male DF-NE DRG transcriptomes was performed using the same filtering parameters as described in previous sections. This comparison led to a gene list of 333 differentially expressed genes when males were experiencing neuroinflammatory relief (Figure 1b) compared to their drug free counterparts. Of these 333 differentially expressed genes, 302 were significantly up-regulated and 31 were significantly down-regulated. The top 25 of these significantly up-regulated and down-regulated genes are featured in Table 7 and Table 8 with the top 10 of each being highlighted in the volcano plot in Figure 8a. 

Enriched biological processes were evaluated with Metascape gene annotation and analysis resource under the express analysis settings. All 333 significantly differentially expressed genes were included in the analysis split into two runs for upregulated gene expression and down regulated differentially expressed genes. The enriched biological processes from the up-regulated gene list are shown in Figure 8a with the two top enriched biological processes being neutrophil degranulation (R-HSA-6798695) and leukocyte migration (GO:0050900). The down-regulated differentially expressed genes influenced by neuroinflammatory relief enriched pathways included neuron projection development (GO:0031175) and cellular response to steroid hormone stimulus (GO:0071383). 

### 2.7. Shared and Unique Gene Expression Changes across Male and Female Neuroinflammation and Relief

**Figure 9:**
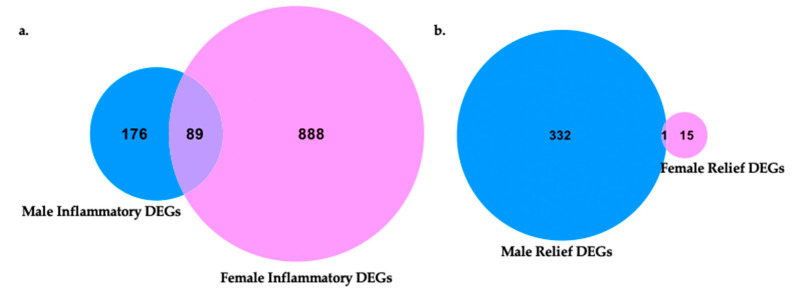
Venn diagrams of differentially expressed genes in each sex and shared genes in male and female neuroinflammation and relief. (**a**) Male and female differentially expressed neuroinflammatory genes compared between sexes for unique characteristics to males or females and those shared between sexes. Within each sex, neuroinflammatory gene sets originated from CCI vs. naïve transcriptome comparisons (female naïve *n* = 3, female CCI *n* = 2, male naïve *n* = 3, male CCI *n* = 3). (**b**) Neuroinflammatory relief gene expression profiles of males and females were compared for shared features and those unique to a sex. Within each sex, neuroinflammatory relief gene sets originated from 10X CXB-NE vs. DF-NE DRG transcriptome comparisons (female DF-NE *n* = 2, female 10X CXB-NE *n* = 2, male DF-NE *n* = 3, male 10X CXB-NE *n* = 3).

Once the DEGs from male and female neuroinflammation and relief had been revealed, they were then compared and are presented in a Venn diagram generated by CLC Genomics Workbench (Figure 9). Again, only genes with an FDR *p*-value ≤ 0.05 and fold change of greater than ±1 are included.

Following the induction of peripheral neuroinflammation by CCI of the right sciatic nerve, there were 977 DEGs in the corresponding female dorsal root ganglia. Of these DEGs, 888 were unique to female neuroinflammation while 89 of them were shared with male neuroinflammation. DEGs distinctive to males from peripheral neuroinflammation included 176 genes (Figure 9a).

In contrast, during 10X CXB-NE driven neuroinflammatory relief from hypersensitivity, male dorsal root ganglia exhibited 333 DEGs while females have 16 DEGs (Figure 9b). In our analysis, males and females shared 1 common significant DEG following treatment, which was *hemoglobin alpha, adult chain 3* (*Hba-a3*). It is interesting to note that using the less stringent *p*-value many more genes fell into each category including 120 genes that were shared between female relief and male relief (Supplementary Figure S2).

These collections of shared and sex associated genes were then examined for changes of expression between sexes following peripheral neuroinflammation and relief for specific genes listed in Table 9. Some of these include *corticotrophin releasing hormone* (*CRH*), which significantly increases RNA expression in both sexes following CCI but is down-regulated only in males following 10X CXB-NE treatment. There is also *activating transcription factor 3* (*Atf3*), which was upregulated in both sexes following peripheral neuroinflammation, but only significantly down-regulated in females following 10X CXB-NE treatment (Table 9). *Atf3* was also analyzed by quantitative PCR of the RNA extracted from the dorsal root ganglia to reveal comparable trends of differential expression (Supplementary Table S3 and Supplementary Figures S3 and S4).

**Figure 10:**
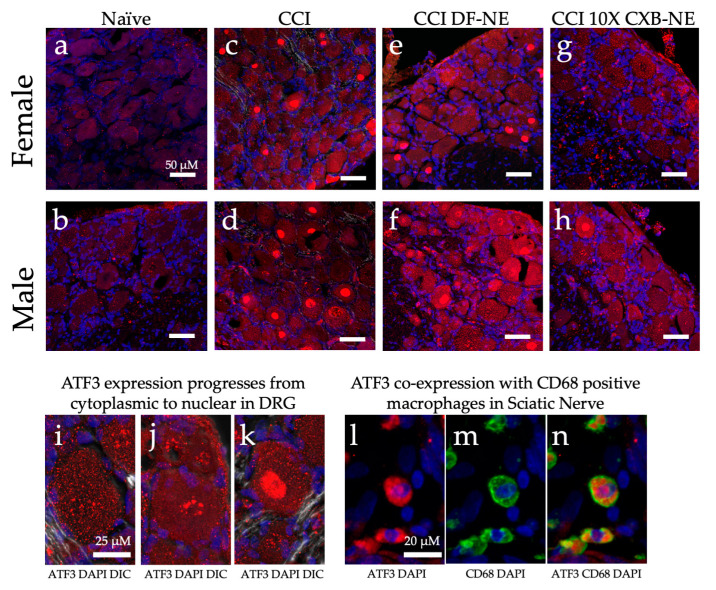
ATF3 protein expression (red) changes in a sex specific manner in the ipsilateral DRG during hypersensitivity (CCI and CCI DF-NE) and with relief (CCI 10X CXB-NE). Tissue retrieved on day 12. Nuclei stained with DAPI (blue). Double stained merge images include DIC transmitted light. (**a**) and (**b**) Non-surgical naïve female and male DRG tissue with low level dispersed ATF3 located throughout the cytosol. (**c**) and (**d**) ATF3 increased expression and condensation in the nucleus following CCI neuroinflammatory hypersensitivity in both female and male ipsilateral DRG. **(e)** and **(f)** CCI treated with DF-NE continue to express high levels of ATF-3 in both cytoplasm and condensation in nuclei for both females and males. (**g**) Disappearance of nuclear condensation of ATF3 following pain-relieving theranostic nanoemulsion with 10X CXB-NE treatment in females. (**h**) Decreased nuclear condensation of ATF3 following 10X CXB-NE treatment in males. (**i**) Cytoplasmic ATF3 localization. (**j**) Perinuclear accumulation of ATF3. (**k**) Condensation of ATF3 in the nucleus. (**l**) Positive ATF3 (red) in macrophages of the sciatic nerve with (blue) DAPI nuclei. (**m**) The same field of view as (**l**), revealing positive staining of macrophages with the anti-CD68 (green). (**n**) Co-localization of ATF3 staining within CD68 positive macrophages within the sciatic nerve.

Further examination of unique gene expression between male and female neuroinflammation and relief was investigated by immunofluorescent staining for the ATF3 protein. While examining the affected dorsal root ganglia recovered from animals on day 12, ATF3 protein was observed in all conditions. Naïve DRG of both males and females appeared to have low levels of this CREB protein dispersed in the cytoplasm (Figure 10a,b,i). Once CCI was performed and neuroinflammation is evident at day 12, ATF3 presence increased and condensation in the nucleus of neurons in the DRG 12 days after the injury (Figure 10c,d). The apparent progression of ATF3 protein accumulation in the nucleus is evident in Figure 10i–k. The CCI treated with DF-NE exhibit a high level of ATF3 protein expression with nuclear condensation. Following 10X CXB-NE treatment, which provided relief from mechanical allodynia, ATF3 expression decreased in both sexes (Figure 10g,h) with greater reduction evident in the female dorsal root ganglia neurons where no nuclear condensation was observed (Figure 10g). Examination of injured sciatic nerve at day 12 reveals some co-localization of ATF3 in CD68 positive macrophages (Figure 10l–n).

## 3. Discussion

This study set out to examine how allodynia induced by peripheral neuroinflammation causes changes in the transcriptome of the peripheral nerve cell bodies and associated cells of the dorsal root ganglia and how RNA expression is changed when this allodynia is relieved by a novel COX-2 inhibiting theranostic nanoemulsion. Here, we show that even though males and females develop quantitively comparable hypersensitivity along the same time course (Figure 1), the gene expression within the affected dorsal root ganglia are distinct. While we also observe that there are some differences in RNA expression evident between individuals of the same sex and treatment group (Figure 3), we resolve statistically significant differences between sexes. For example, of more than 1000 genes that exhibited differential expression in both males and females experiencing neuroinflammation, only 89 are shared between the two sexes (Figure 9a). Furthermore, with 10X CXB-NE providing similar mechanical hypersensitivity relief in both males and females, differences in changed gene expression are yet again evident between the sexes. Exhibiting the same behavior does not require the same gene expression. Examining gene expression in this way is beginning to reveal some of the fundamental similarities and sex differences in the cellular response to injury and differences in response to pain-relieving drug therapy. These are crucial findings given that there still no sufficient treatments for chronic neuroinflammatory pain and further highlights the need for a deeper understanding of the sex differences in these biological processes.

### 3.1. Mechanical Allodynia Relief from NSAID 10X CXB-NE 

In order to evaluate the transcriptomic changes associated with peripheral neuroinflammation and the relief from a COX-2 inhibiting theranostic nanoemulsion, CCI of the right sciatic nerve was performed for both sexes. Quantifying mechanical allodynia by von Frey technique reveals that as compared to the naïve and base-line response of 16–17 g force for withdrawal for both male and females, both sexes develop comparable hypersensitivity averaging around 5 g of force as the 50% withdrawal threshold (Figure 1b,c). This means that following identical surgeries, both sexes experienced the same levels of neuroinflammatory pain. Furthermore, once a single dose of the COX-2 inhibiting theranostic nanoemulsion (2.4 mg/kg) was administered on day 8 (Figure 1a), both males and females received similar multi-day relief from pain-like hypersensitivity (Figure 1b,c). In fact, with this 10X CXB-NE, by day 10 (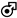 *p* = 0.0919, 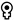 *p* = 0.3908) and day 12 (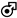 *p* = 0.8712, 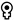 *p* = 0.8643) there is no longer a significant difference in behavior between drug treated animals of either sex and their baseline or naïve behavior. Recall that a different 1× CXB-NE formulation (0.24 mg/kg) presented to CCI male and female rats provided complete relief for males, but only partial relief for females [11] and that the 1× CXB-NE delivered to CFA mice also resulted in sexually dimorphic relief [27]. So, with the 10X CXB-NE, the higher level of celecoxib per nanodroplet could be influencing COX-1 activity in females. This model is consistent with other researcher’s observation that in a CFA inflammatory pain model using COX-1 and COX-2 knockout male and female mice, a difference in the involvement of these enzymes was detected between the sexes [28]. Chillingworth and colleagues [28] saw that male inflammatory relief was most effectively achieved by COX-2 inhibition while females appeared to have an additive effect based on the involvement of both COX-1 and COX-2 in the development of their inflammatory pain [28]. Given that celecoxib has a slight selectivity for COX-1 [29,30,31], this could mean that COX-1 and COX-2 are now effectively attenuated in the females and thus, complete relief similar to that seen in males can now be experienced. Given that males already have complete relief, more celecoxib can’t influence their behavior any further.

With the male and female rats experiencing similar levels of mechanical allodynia (CCI and DF-NE) and similar levels of relief (10X CXB-NE) on the day of tissue recovery, the assessment of the relative number of infiltrating CD68 positive macrophages reveals equivalence between the sexes (Figure 2). However, even though the male and female behavior is the same and the degree of inflammation runs in parallel for each, the analysis of RNA transcription during neuroinflammation and during relief derived from celecoxib reveals significant, and fundamental differences exist between the sexes. While we have previously observed a sex difference in relief from CCI using a 1× celecoxib theranostic nanoemulsion treatment [11], that study used a lower dose of celecoxib per nanoemulsion droplets. Here, the higher drug loading in this new formulation completely closes the gap in relief with both sexes experiencing quantitatively identical mitigation of behavioral hypersensitivity. Previously demonstrating that there is a sex difference in the degree of relief from the lower celecoxib dose unequivocally revealed that males and females are responding to pain and pain-relief differently [11].

While others have begun to examine the sex differences in neuroinflammatory induced pain [14,15,16,17,18] or baseline sex differences in the naïve DRG [16], this is the first study examining the sex differences in the DRG transcriptome resulting from neuroinflammatory relief by an effective COX-2 inhibitory treatment. 

### 3.2. Male Transcriptome Changes in the DRG following CCI 

Following peripheral nerve injury, the associated ganglia are impacted, and transcriptional changes occur due to interactions with glial cells, immune cells, and adjacent neuronal cells [20,32]. This crosstalk impacts neuronal hypersensitivity leading to the perception of pain by the central nervous system [32]. We have previously seen immune cell infiltration in the DRG [24], and the findings in this study suggest that following the CCI injury, immune cells are present in the DRG and may exert an impact on transcriptional regulation of the DRG (Figure 6b,c). Specifically, genes such as *neuropeptide y* (*NPY*), *thrombospondin-1* (*THBS-1*), *complement factor h* (*CFH*), and *activating transcription factor 3* (*Atf3*) are up regulated following neuroinflammation in the male DRG (Table 3). These genes ultimately contribute to the production of proteins that influence the inflammatory response [33,34,35,36]. 

Some genes such as *NPY* and *THBS-1* are known to induce phagocytosis of damaged cells by macrophages and neutrophils respectively [34,35]. *NPY* is also well known to affect cell migration, cytokine secretion, and cell adhesion [35]. Other genes such as *CFH* have been shown to prevent the inflammatory response from overreacting and protect un-damaged cells by keeping the complement system turned off [36]. *Atf3*, a member of the cAMP response element binding (CREB) protein family, has been shown to be both a repressor and an activator of the immune response depending on the circumstances [33]. Other studies have also seen ATF3 to be significantly upregulated following peripheral nerve injury, indicating its importance in maintaining this inflammatory balance in the neuroimmune response [18]. Interestingly, *Atf3* induction appears to be selective for compounds or insults that lead to nerve injury and not agents that cause inflammation and a simple increase in nerve activity such as CFA [37,38].

Overall, stable changes in gene expression following peripheral neuronal injury can be seen to be maintaining an allostatic balance [39] while increasing the inflammatory response to facilitate tissue repair and regeneration. 

Because of this damage, down regulation of myelination and cytoskeleton dynamics during Wallerian degeneration is known to occur following peripheral nerve injury [40]. During Wallerian degeneration, injured peripheral nerves signal to phagocytic immune cells and Schwann cells to de-differentiate and contribute to the phagocytosis of dying and damaged cells. During this process, the damaged axon retracts distally, and myelination significantly decreases [40]. Genes that contribute to these activities are seen to be enriched in the down regulated processes in this study (Figure 6c) as well with genes involved in myelination, such as *early growth response 2* (*EGR2*) and *zinc finger and BTB domain containing 16* (*ZBTB16*), both being significantly down regulated following CCI of the right sciatic nerve (Table 4). EGR2 is a DNA binding transcription factor known to regulate Schwann cell myelination by regulating other genes involved with myelination [41]. ZBTB16 is primarily known to be involved with neuronal differentiation; however, a recent study found it to be detrimental to neocortical myelination when knocked out of oligodendrocytes in the central nervous system [42]. Cytoskeleton genes such as *spectrin beta, non-erythrocytic 5* (*SPTBN5*), who’s role is in cytoskeleton dynamics and signaling, is also seen to be down regulated [43]. Interestingly, cytoskeleton elements seen in muscle and muscle contraction are also found to be down regulated in the DRG following injury (*Myosin heavy chain 2* (*myh2*) [44], *troponin C2* (*Tnnc2*) [45], and *Troponin T3* (*Tnnt3*) [46]). This could indicate multiple uses for these cytoskeleton components not only in muscle contraction but also in neuronal remodeling. 

### 3.3. Female Transcriptome Changes in the DRG following CCI 

Our understanding of the development of female neuropathic pain is just beginning to evolve. In this study and the research by others is revealing both similarities and a differences in the transcriptomes of the DRG for males and females experiencing neuropathic pain [18]. As with male neuroinflammation, female neuroinflammation exhibits up-regulation of immune modulators such as *NPY* [35] and *Atf3* [33] (Table 1). This increase in *Atf3* in both sexes following nerve insult is in agreement with other transcriptional studies looking at sex differences following peripheral nerve injury [18,20]. However, we see a higher fold change of RNA expression in females (7.8) compared to that seen in males (4.1) (Table 1 and Table 3). This heightened gene expression in females when compared to males is also seen with *NPY,* where animals that have undergone CCI have a 5.8-fold change in males when compared to naïve, but a 12.4-fold change in female neuroinflammation. In addition, other up-regulated enriched pathways in females following CCI include increased cytokine signaling in the immune system and microtubule processing (Figure 5b). 

Some of these genes that are up-regulated and exhibit involvement in microtubule processing include *Fibronectin leucine rich transmembrane protein* (*FLRT3*) and *growth associated protein 43* (*GAP-43*) (Table 1). *FLRT3* has previously been seen to be upregulated in the DRG following peripheral nerve injury and modulates neuron growth [47]. While *GAP-43* is expressed in growing neurons, once retrograde axonal transport has been interrupted it is associated with neuron regeneration [48]. The increased gene enrichment in microtubule processing in females is contradictory to what was observed in male neuroinflammation, where a down regulation of myelination and decreased cytoskeleton dynamics were seen to be enriched, corresponding with Wallerian degeneration. This is not to say that Wallerian degeneration is not affecting the female DRG, but perhaps this process of clearing cellular debris is occurring differently in the female neuropathy than in the male. Further histological examination will be required to resolve this.

### 3.4. Macrophage Associated COX-2 Inhibitory Effect Causes Transcriptional Differences in the Associated Male DRG

While examining the transcriptomic changes uniquely associated with male mechanical allodynia relief, several enriched pathways come to light, with one being an increased expression for neutrophil degranulation (Figure 8b), which suggests that neutrophils may be infiltrating the DRG at this point. Neutrophils, under normal circumstances, have a role in clearing cellular debris through phagocytosis, degranulation, and NETosis [49]. Once differentiated in the bone marrow, neutrophils bear the CXCR2 motif and are held in the bone marrow by the CXCL12 ligand until signaled for by granulocyte colony stimulating factor (G-CSF) [50]. Macrophages at the injury are signaled to release G-CSF by the calcium binding proteins *S100A8* and *S1009A*, which in turn initiate the neutrophil chemotaxis and then support the degranulation of enzymes such as myeloperoxidase (MPO). Our study shows upregulation of all of these components including *CXCR2*, *CXCL12*, *S100A8*, *S100A9*, and *MPO*. Neutrophil’s presence is typically associated with acute inflammation, and their function is normally complete within 24 h and then they are eliminated by macrophages [49]. However, some recent studies have found that neutrophils can exhibit anti-inflammatory properties bearing the arginase 1 (*Arg1*) protein and have been termed N2 type neutrophils [51]. Another study found that immaturely delayed neutrophils expressing Arg1 also had enriched *Tgfb1* and excreted higher levels of nerve growth factor (NGF) [52]. Our study also reveals a 2.4-fold increase of *Arg1* gene expression, 2.5-fold increase in *NGF*, and a 1.4-fold increase in *Tgfb* suggesting that with this gene expression signature, N2 anti-inflammatory neutrophils could also be present in the DRG. While it needs to be independently confirmed, it is interesting to consider that the relief in neuroinflammatory pain allows for a second set of neutrophils with the N2 phenotype to enter the DRG. 

A study by Parisien et al. [53] looked at how the neutrophil response plays a role in developing and reducing neuropathic pain. They examined human data from patients with acute lower back pain and tracked them over a three-month period. Peripheral blood samples showed those with sustained pain had no differentially expressed genes when comparing RNA expression from their acute visit to the three-month time point. However, those experiencing relief had differentially expressed genes related to neutrophil involvement such as *S1008A* and *S1009A.* Parisien and colleagues [53] also correlated the use of NSAIDs to decreases of this early neuroinflammatory response, patients were twice as likely to develop long term neuropathic pain [53]. This was interpreted as a reduction of neutrophils early on, which typically function to clear cellular debris, and provide for neuronal regeneration, while influencing the local inflammatory response. To confirm that the early use of NSAIDs can induce higher risk of chronic pain, and that it can be rescued by *S1008A* and *S1009A* proteins, they tested mice that underwent CCI and CFA inflammatory induction. Each were treated with the NSAID diclofenac for the 6 consecutive days following injury. Once treatment wore off, the duration of mechanical allodynia was significantly greater in those treated with diclofenac in CFA models. Furthermore, in the CCI model, the NSAID treatment was followed by prolonged duration of allodynia [53]. They then saw that the prolonged hypersensitivity due to NSAID treatment could be reversed by injections of neutrophil specific proteins *S100A8* and *S100A9* [53]. Our study presented here is in agreement with this Parisien et al., where we find that the upregulation of *S100A8* and *S100A9* is correlated to a reduction in neuropathic pain in males treated with 10X CXB-NE. It is important to recognize a significant difference in these two studies is that the Parisien et al., looks at 6-day dose of NSAIDs at the onset in the inflammatory insult, where we explored a response to the use of NSAIDs presented for the first time 8 day post-injury.

Down regulation of some enriched biological pathways are also present when mechanical allodynia was relieved including regulation of system processes (Figure 8c). One of the genes enriched in the regulation of system processes was *corticotrophin releasing hormone* (*CRH*). *CRH* was up regulated in male neuroinflammation (33.5-fold) and significantly down regulated only with male neuroinflammatory relief (−21.6-fold). Although *CRH* is primarily studied in the central nervous system, recent investigations of the peripheral nervous system have shown it to be analgesic [54]; however, these studies look at administration of corticotrophin releasing factor systemically [55,56] rather than endogenous CRH function. Although our findings are counterintuitive to what these other studies have seen, Reinhold and colleagues using the CCI model saw that the most changed gene expression following DRG damage due to CCI was *CRH* [57]. Our findings are in agreement with this, showing the *CRH*’s function endogenously may differ from its systemic effect. Although *CRH* is seen to also increase 34.5-fold following CCI in our study, it is only the males that have a significant decrease in *CRH* following mechanical allodynia relief; thereby, revealing an unknown pharmacodynamic sex difference.

### 3.5. Macrophage Associated COX-2 Inhibitory Effect Causes Transcriptional Differences in the Associated Female DRG

Females experiencing neuroinflammatory relief were affected very differently than males, revealing only 16 significant DEGs compared to 333 DEGs in males (Figure 9) (limit of FDR *p*-value ≤ 0.05 and fold change of greater than ±1); however, there were quite a few genes that were upregulated during mechanical allodynia and reduced when mechanical allodynia was relieved. Some of these genes include *Atf3*, *Transcription factor JUN* (*JUN*), and *VGF nerve inducible factor* (*VGF*).

VGF is a peptide that is increased with nerve growth factor and highly expressed in the peripheral and central nervous system following neuropathy [58]. Some studies administrating VGF peptide intrathecally have also seen that it is associated with peripheral mechanical hypersensitivity [59]. Our study, as well as others have seen *VGF* to be transcriptomically increased in both the male and female DRG following peripheral neuroinflammatory induction [18]. However, here we see that only in the female DRG is VGF significantly reduced following 10X CXB-NE treatment (−1.6-fold). Soliman and colleagues have written an extensive review about VGF and its increase in expression being undoubtedly related to inducing neuropathic hypersensitivity [58]. They suggest that targeting VGF derived peptides may be a possible therapeutic for pain relief [58]. Our studies support this claim given that when VGF is reduced in females, mechanical hypersensitivity follows suit.

Another interesting enriched biological process is the PID ATF2 Pathway with *Atf3*, *JUN*, and *Sema6a* following within it. *Atf3* will be extensively discussed in the next section; however, *JUN* also has distinct roles in regulating neuroinflammatory pain. *JUN* is the gene encoding the c-Jun transcription factor highly associated with c-Jun *N*-terminal kinase (JNK). The JNK pathway’s typical role is in stress signaling within the cell. It has been observed to be upregulated following spinal nerve ligation in both the spinal cord and DRG and has a linked role in maintaining neuropathic hypersensitivity [60]. When an inhibitor to JNK was administered intrathecally via osmotic pump, a reversal of mechanical allodynia was observed [60]. Here, in females, we also see that *JUN* is significantly down regulated when female neuropathic pain is relieved by 10X CXB-NE. This suggests that the involvement of the JNK pathway can both relieve pain [60], but also is reduced when neuroinflammatory pain is reduced.

### 3.6. CREB Protein Involvement in the Sex Differences of Neuroinflammation and Mechanical Hypersensitivity Relief

One of the more interesting finds in this study was the involvement of the CREB protein ATF3 and the sex differences that were evident in the gene expression between components of the pathway during neuroinflammation and relief. *Atf3* is significantly upregulated in the DRG of both sexes following neuroinflammation, but only significantly down-regulated in females following treatment. 

**Figure 11:**
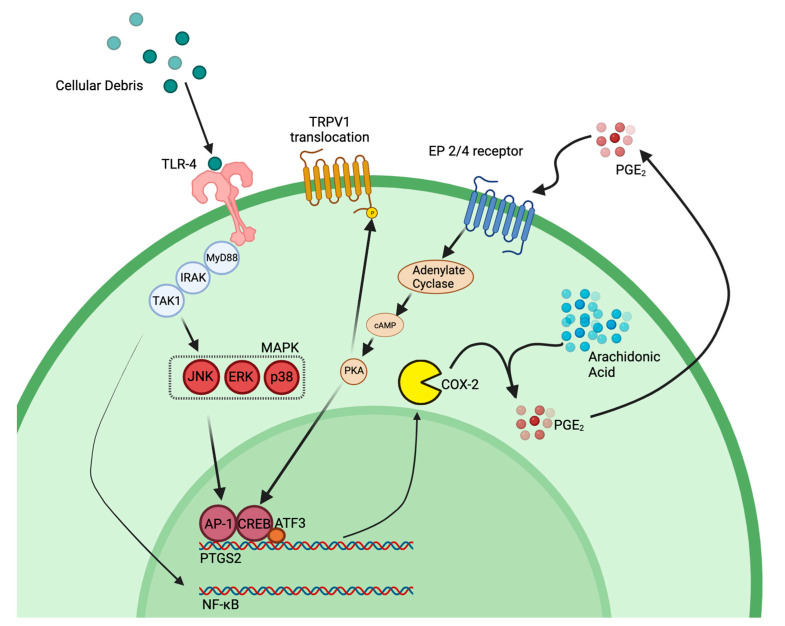
COX-2 feedback loop transcriptionally regulated by ATF3. TLR4 is activated by cellular debris from neuronal injury as well as certain cytokines, which then activates parts of the MAPK signaling cascade as well as NF-κB. Production of PGE_2_ provides a positive feedback loop to support continued production of prostaglandin, which further support increased immune cell infiltration and activation. PKA, which is activated through PGE_2_ binding to its EP4 receptor, can phosphorylate TRPV1 inducing translocation of the receptor to the cell membrane; thereby, increasing neuronal hypersensitivity. Model influenced by our research and multiple citations [33,62,65,66]. Figure made with BioRender.

ATF3 is a member of the CREB protein transcription factor family, which can be activated during an immune response by ER stress, cytokines, chemokines, hypoxia, DNA damage, and growth factors [61]. In turn, CREB proteins can lead to the activation of different toll-like receptors (TLRs) [33]. For example, *Atf3* activation in a macrophage is known to down-regulate the inflammatory response under normal conditions by regulating Th17 T cells, decreasing inflammatory cytokines (IL-6, IL-12b, and IL17) and promoting macrophage survival [33]. Interestingly, one study found that not only does the ATF3 protein regulate immune response, but it also is able to bind to the promotor of the *Ptgs2* gene, which transcriptionally regulates the COX-2 enzyme [62]. We find that some of the CD68 positive macrophages in the injured sciatic nerve co-express ATF3 (Figure 10j,k,l). Because *Ptgs2* is involved in a regulatory feedback loop with its product prostaglandin E_2_ [63], it is intriguing to consider how attenuating the activity of the COX-2 enzyme in a macrophage with our novel COX-2 inhibiting theranostic nanoemulsion may play a role in influencing gene transcriptional changes, specifically with the CREB pathway components (summarized in Figure 11). Additionally, PGE_2_ binding to its EP4 receptor leads to the activity of protein kinase A (PKA). PKA has been shown in other studies to phosphorylate transient receptor potential vanilloid 1 (TRPV1) thereby targeting it to the cellular membrane. TRPV1 is known to increase neuronal hypersensitivity by reducing firing thresholds; thus, increased translocation of this receptor leads to increased neuronal hypersensitivity and pain [64]. 

ATF3 has been previously shown to also express unique functions in neuronal cells. Following nerve injury, *Atf3* responds as an immediate early gene with transcription taking place to help regulate the neuronal response to inflammation [67]. For example, assessing skin incision injury on female rats revealed that *Atf3* was upregulated in the DRG; DiI, placed at the site of injury facilitated the identification of the specific neuronal cell-bodies that innervated the injury [68]. The ATF3 upregulation persisted for weeks following that injury [68]. Our results show that ATF3 transcription and protein expression correlates with pain and pain relief, suggesting increased expression of *Atf3* could be involved in transcriptionally altering the nerve cells in ways that contribute to their hypersensitivity. 

Mechanistically, our COX-2 inhibitor therapeutic may function indirectly to decrease *Atf3* production in neurons (Figure 10) by decreasing overall PGE_2_ in the cellular milieu, thus attenuating the PGE_2_ feedback loop (Figure 11). 

In the naïve DRG in both sexes, low levels of the ATF3 protein can be seen located in the cytoplasm of neuronal cell bodies (Figure 10a,b). Following neuroinflammatory induction, RNA-seq detected significant increases in *Atf3* with a 7.8-fold increase in the female DRG and a 4.1-fold increase in the male DRG when compared to the respective naïve (Table 2 and Table 3). Our observation is consistent with a similar study that also saw this sex difference following CCI induction where females had increased *Atf3* expression compared to males [18].

In injured tissue, ATF3 protein can be seen to progressively congregate in the nucleus of DRG neurons (Figure 10j) until it is densely located within (Figure 10k). We observed the highest number of neurons with ATF3 dense nuclear stains in those samples experiencing neuroinflammatory induced allodynia (Figure 10c–f), and reduced levels in those who received 10X CXB-NE treatment (Figure g,h). Similarly, RNA-seq detected a significant decrease (2.1-fold) in *Atf3* expression in females treated with 10X CXB-NE while an increase in *Atf3* (2.0-fold) was evident in treated males. This difference is again seen in our data for other CREB pathway components downstream of *Atf3* such as *Jun,* with both males and females exhibiting increased levels of expression following neuroinflammation (1.6-fold in females and 1.3-fold in males), but again, only females having significantly decreased expression of *Jun* following 10X CXB-NE treatment (−1.3-fold) while males again have a slight increase in expression of *Jun* (1.3-fold). *IL-6*, which is also downstream in the CREB response pathway, shows this similar pattern but at a more amplified manner in females (24.0-fold increase) when compared to males (3.8-fold). IL-6 is known to prevent *FoxP3* expression in T helper cells [69]. It is interesting to speculate that perhaps this change in IL-6 expression in females may in fact contribute to a pro-inflammatory role by not allowing T helper cells to differentiate into regulatory T cells. Following treatment, females again have decreased *IL-6* regulation (−4.6-fold) when comparing 10X CXB-NE to DF-NE, while males again have a slight increase in expression (2.0-fold).

## 4. Conclusion

Given that neuropathic pain currently has no effective long-term treatment, and that female neuropathic pain has been under studied, the need to evaluate the differences between male and female neuroinflammation and relief is warranted. This study elucidates some of these differences in the dorsal root ganglia transcriptome of male and females experiencing mechanical hypersensitivity and also while experiencing relief. Here we show that male and female rats who have undergone CCI of the right sciatic nerve experience similar levels of mechanical allodynia while a novel COX-2 inhibiting theranostic nanoemulsion is able to provide extended relief equally to both males and females to a similar degree. This means that the transcriptomic differences seen between the sexes are not due to different levels of pain or relief from pain, rather that they are due to sexual dimorphic expression of regulatory pathways. Some genes of interest playing unique roles in male neuroinflammation and relief include *CRH* as well as genes associated with neutrophil degranulation. While in females, some of the key genes include *Atf3*, *JUN*, and *VGF*.

The study presents a few remaining questions that warrant further investigation. For example, the 10X CXB-NE does not seem to be showing a different response in males as compared to earlier published work with 1X CXB-NE [11]. Once behavioral relief to baseline is achieved by COX-2 inhibition in males, increasing the dose of drug doesn’t change behavior. However, in females, we saw marked improved response as compared to 1× CXB-NE [11]. This implies that there are additional effects of celecoxib in females that have yet to be determined. Comparable relief between sexes that is achieved with increased drug loading of the theranostic opens new questions. 

Nevertheless, this study reveals that even when males and females experience similar levels of neuropathic pain and that when they achieve comparable relief from a macrophage targeted COX-2 inhibition by a theranostic nanoemulsion, the basic underlying biology of how these behaviors occurs in each sex differs. This means, there is an opportunity to further investigate sex specific targets for new therapeutic interventions.

## 5. Materials and Methods

### 5.1. Animal Use and Ethics

The following experiments were carried out at Duquesne University, Pittsburgh, PA, USA, in compliance with the recommendations in the Guide for The Care and Use of Laboratory Animals and the regulations of the Institutional Animal Care and Use Committee (IACUC) under the approved protocol #2009-07 as well as the animal care and use review office (ACURO) for the Department of Defense (DM190725.e001, approved 21 December 2020). 

Male and female Sprague Dawley rats were purchased through Hilltop Lab Animals, Inc. (Springdale, PA, USA). Once received, animals were acclimated for 1 week and then handled for 1 week by the animal behavioralist prior to use. Prior to the experiment beginning, animals were socially housed; animals were individually housed once baseline von Frey behavior began (2 days prior to surgery). Individual housing was done to keep rats from opening one another’s incisions post-surgery. Animals were kept on a 12:12 h light-dark cycle with access to food and water *ad libitum*. Special diet (Research Diets, Inc., New Brunswick, NJ, USA; Cat # AIN-93G) was used to avoid interference of autofluorescence during near infrared imaging of the theranostic nanoemulsion. The number of animals used was based on a power analysis of our previous studies. For this study, the number of animals per condition is defined first in the behavioral assessment. Using a power of 80% and an alpha value of 0.05, it was determined that a total of 4 animals per condition was needed for these studies. Here, females *n* = 5 for naïve, *n* = 6 for CCIs, *n* = 7 for DF-NE, and *n* = 7 for 10X CXB-NE; for males, the inclusion for behavior was *n* = 5 for naïve, *n* = 4 for CCI, *n* = 7 for DF-NE, and *n* = 7 for 10X CXB-NE. Animals were then dedicated to either RNAseq analysis or immunohistochemical assessment as noted in the relevant figures.

### 5.2. Up-Down Von Frey Behavior

Cohorts of all male or all female rats were tested separately for hypersensitivity with the von Frey technique. In each case, behavioral analysis was assessed every other day before and following surgery as outlined in Figure 1a. Behavioralist was blinded to any treatment received. Rodents were first acclimated for 30 min in individual plexiglass containers with mesh floors. Once testing began, the left and right hind paws were probed with calibrated von Frey filaments (Stoelting Co., Wood Dale, IL, USA, Catalog # 58011). Calibrated filaments (1.202 g, 1.479 g, 2.041 g, 3.630 g, 5.495 g, 8.511 g, 15.136 g) were applied to the hind paw at a 30-degree angle and for 3 s to look for positive (quick lift, flick, vocalization) or negative responses. If no response, the next largest filament would be used. If positive response, the next smallest filament would be used. This pattern continued until 4 responses were collected after the change in response such as used previously [11,23,24,70,71]. No less than 30 s separated probing of the same paw twice. The gram force of the 50% withdrawal threshold was then calculated using Chaplan’s 50% withdrawal threshold formula [70].

### 5.3. Chronic Constriction Injury (CCI)

Neuroinflammation and mechanical hypersensitivity was induced via CCI as described by Bennet and Xie [72] and as previously done in our laboratory [11,23,24]. Surgery was performed under isoflurane anesthesia. The surgical area was first clipped of hair and sterilized with iodine and alcohol. An incision through the skin was made just beneath the right femur bone. The biceps femoris and gluteus superficialis were then bluntly separated to expose the right sciatic nerve. The sciatic nerve was freed from surrounding connective tissue and 4 sutures (McKesson 4-0 Chromic Gut Sutures, Irving, TX, USA, Catalog # S635GX) were loosely tied around the sciatic nerve approximately 1 mm apart. Muscles were closed with sutures and skin was stapled close with 9 mm autoclips (Braintree Scientific, Braintree, MA, USA, Catalog # NC9281117). 

### 5.4. COX-2 Inhibiting Theranostic Nanoemulsion

Nanoemulsions for this study were manufactured following previously reported procedures [11,23,73,74,75], with some minor modifications. Briefly, the M110S microfluidizer (Microfluidics Corporation, Westwood, MA, USA) interaction chamber was iced for 1-h prior manufacturing and all processing steps performed while the chamber was on ice to prevent dye and/or drug degradation. Celecoxib (CXB) was pre-dissolved in oils and co-solubilizers overnight, as reported earlier [74], to achieve final concentration of 2.4 mg/mL in the 10× CBX-NE. For drug-free (DF-NE), nanoemulsions were processed the same way with omitting the drug incorporation step. Imaging agents, near infrared fluorescent (NIRF) dye (DiR) and perfluoro-15-crown-5-ether (PCE) were added as reported previously [74,76]. Nanoemulsion pre-mix included, drug, dye, hydrocarbon oils, PCE and solubilizers, was vortexed on high prior to adding surfactant mixture [74] pre-dissolved in 1× PBS at final concentration of 5% *w*/*v*. The pre-emulsion, formed by brief vortexing, was poured into the microfluidizer M110S inlet reservoir and processed at an operating liquid pressure ∼15,000 psi. All nanoemulsions were sterile filtered through 0.22 μm filter, stored in at 4 °C prior use. The resultant nanoemulsions had the same droplet size distribution as previously reported (130–140 nm) [11,23,74]. 10X CXB-NE and DF-NE characterizations (droplet size distribution, fluorescence comparison between the two formulations, stability, and cell viability) are summarized in supplementary Figure S5.

### 5.5. Tail Vein Injection

The 10X CXB-NE (celecoxib) or DF-NE (drug free) was delivered via tail vein injection on the 8th day following surgery as previously described [77]. The theranostic nanoemulsion is tagged with the near-infrared dye DiR (1,1′-dioctadecyl-3,3,3′,3′-tetramethylindotricarbocyanine iodide). Accuracy and quality of the tail vein injection can therefore be assessed using live animal near infrared fluorescent imaging. Briefly, under anesthesia, the rat’s lateral tail veins were dilated by placing the tail into a warmed water bath. A 27 G needle was inserted into the lateral tail vein, and blood flashback was observed. Slowly, 300 µL of either 10X CXB-NE (55.5 micrograms of CXB) or DF-NE was injected into the tail vein [11,23,24]. The same dosage of celecoxib was used for all animals [11]. The injection quality was then assessed using the LI-COR Pearl Imager (LI-COR Biosciences, Lincoln, NE, USA). A quality injection exhibits clearance of the dye from the site of injection leaving little to no near infrared fluorescent signal in the tail. Any animals with 10X CXB-NE or DF-NE remaining in the tail from a ruptured vein or subcutaneous injection were removed from the study.

### 5.6. Estrus Cycle Tracking

As we have done previously [11] the female estrus cycle was tracked for five consecutive days (days 8–12 post-surgery) in order to track the estrus cycle and establish where in estrus cycle the individual was when receiving treatment. During this procedure, females were briefly anesthetized with isoflurane, vaginal canals were flushed with saline, and collection was allowed to dry on a slide. Once dry, slides were fixed with 100% methanol and stained with 5% Geisma stain for 20 min. Slides were then rinsed briefly with water and allowed to dry before mounting with Permount (Thermo Fisher Scientific, Waltham, MA, USA, Cat # SP15-100). Using Cora and colleague’s description of the estrus cycle [78], each female’s cycle was established. 

### 5.7. Dorsal Root Ganglion (DRG) Collection

Rats were euthanized on the 12th day following surgery at the peak of relief from their mechanical hypersensitivity and the control rats (Figure 1b,c). Rats were euthanized by CO_2_ asphyxiation, and the right L4 and L5 DRG were immediately dissected. DRG that were to be sequenced were placed into RNA*later*^TM^ stabilizing solution (Invitrogen, Waltham, MA, USA, Catalog # AM720) and solution was allowed to permeate tissue for 24 h at room temperature. The RL4 and RL5 DRG of the same animal were then combined into a new tube and stored at −20 °C until shipment to Qiagen for RNA extraction, library preparation, and sequencing. 

DRG that were to be used for immunohistochemistry were placed into 4% paraformaldehyde (PFA) in 1× PBS solution for 24 h at 4 °C. DRG were then placed in 30% sucrose solution in 1× PBS and kept at 4 °C. Tissues were embedded in optimal cutting temperature (O.C.T.) compound and 20 µm frozen sections were collected on gelatin coated slides.

### 5.8. Sample Preparation

RNA isolation was performed under contract by Qiagen as follows; RL4 and RL5 DRG were homogenized for RNA extraction using the RNeasy Plus Universal Mini Kit (Qiagen, Hilden, Germany). In order to include small RNAs, manufacturer instructions for the kit were followed using the Qiagen protocol, Purification of Total RNA Containing miRNA. 

### 5.9. Library Preparation and Sequencing

Approximately 400 to 500 ng of extracted RNA was first heat fragmented, and then separated from any unwanted ribosomal RNA (rRNA) using the QIAseq FastSelect rRNA and globin deletion kit (Qiagen, Hilden, Germany). Samples were quality controlled by checking the RNA integrity number (RIN^e^) gathered from the Agilent D1000 ScreenTape System (Agilent Technologies, Santa Clara, CA, USA). Using the QIAseq Stranded Total RNA Library Kit (Qiagen, Hilden, Germany), library preparation was done. Briefly, sequencing adapters were added, and cDNA was amplified using PCR. All libraries were pooled in equimolar concentrations. Following the manufacturer’s protocol, library pools were clustered on the surface of a flow cell before sequencing on a NextSeq High Output flow cell (Illumina Inc., San Diego, CA, USA) instrument (2 × 75, 2 × 8). 

### 5.10. Bioinformatics Analysis of RNA-Seq Data (Mapping and Differential Expression Analysis)

RNA-seq fastq data files were received by the authors from Qiagen and imported into CLC Genomics Workbench 22.0.2 (QIAGEN, Hilden, Germany). CLC was then used for quality assessment, alignment with the *Rattus norvegicus* genome (mRatBN7.2.106), heatmap comparisons, differential expression analyses, volcano plots, and Venn diagram comparisons of the differentially expressed genes.

Mapping was done under the CLC Genomics Workbench default settings in the forward direction. Genes included in most-mapping analyses had an adjusted *p*-value, false discovery rate (FDR), of ≤0.05 and a fold change greater than ±1. Trimmed mean of M (TMM) normalization is internally used for differential expression, heatmaps, and principal component analyses. 

To identify genes that were regulated by neuroinflammation (CCI vs. Naïve) or relief (10X CXB-NE vs. DF-NE) in each sex, differential expression analyses were performed. The gene sets that were differentially changed by neuroinflammation or relief were then compared between sexes as shown with Venn diagrams to determine which genes are shared or distinct to a sex under these conditions. 

### 5.11. Enrichment Analysis

Metascape (metascape.org) was used for enrichment analyses. Metascape is updated monthly and incorporates Gene Ontology (GO) processes, Kyoto Encyclopedia of Genes and Genomics (KEGG) pathways, Reactome gene sets, Cytoscape and NCBI as well as other publicly available resources all incorporated into one platform [79]. This allows for reduced redundancy among ontology terms. 

Up or down-regulated gene sets of differentially expressed genes due to neuroinflammation or relief in each sex were then imported into Metascape using the express analysis version with the default settings. Enrichment heatmaps and protein interaction networks were then visualized for interpretation.

### 5.12. cDNA Conversion and RT-qPCR Validation

Purified total RNA returned from Qiagen was converted to cDNA using the Verso cDNA conversion kit (ThermoFisher Scientific, Waltham, MA, USA; AB-1453/A). RNA (500 ng) conversion was performed at 42 °C for 1 h and then inactivated for 2 min at 65 °C. cDNA conversion was confirmed to be successful via PCR using GAPDH primers and visualization on an agarose gel.

Real time quantitative PCR (RT-qPCR) was done to verify gene expression changes in a subset of genes (*Atf3* and *Sema6a*). Maxima SYBR Green/ROX qPCR Master Mix (2×) (ThermoFisher Scientific, Waltham, MA, USA, Catalog # K0221) was used on the Step One Plus qRT-PCR Instrument (Applied Biosystems, Waltham, MA, USA). Primers are listed in Supplementary Table S3. Briefly, 250 ng of template DNA was used in a 25 µL reaction. Following the manufacturer’s protocol, 40 cycles were run under the two-step reaction protocol. Supplementary Figures S3 and S4 show a summary of the qPCR results.

### 5.13. Immunofluorescent Staining and Confocal Imaging

Fluorescent staining was performed using a rabbit anti-ATF3 primary antibody (Millipore Sigma, Burlington, MA, USA; Cat # HPA001562) at a 1:500 dilution in combination with Alexa Fluor^TM^ 555 donkey anti-rabbit poly-clonal antibody (ThermoFisher Scientific, Waltham, MA, USA; Cat # A31572) secondary antibody. CD68 macrophage detection was done using a mouse monoclonal IgG1 anti-CD68 antibody (Bio-Rad, Hercules, CA, USA; Cat # MCA341R) at 1:500 dilution in combination with Alexa Fluor^TM^ 488 Goat anti-mouse poly-clonal antibody (ThermoFisher Scientific, Waltham, MA, USA; Cat # A21121) following our standard protocols [11].

Briefly, slides were fixed in 4% paraformaldehyde for 15 min and then washed in 1× PBS. Next, slides were permeabilized in 0.3% Triton X-100 for 15 min, washed, and then blocked in 5% normal donkey serum (NDS) for 1 h at room temperature. Primary anti-bodies were then diluted in 5% NDS and allowed to incubate on the slides over night at 4 °C. The next day, slides were washed, and secondary antibody diluted into 5% NDS was incubated on the tissue for 2 h at room temperature. Slides were nuclear stained using ProLong^TM^ Gold anti-fade reagent with DAPI (Invitrogen, Waltham, MA, USA; Cat # P36935).

Images were acquired on a Nikon Ti_2_ Eclipse inverted A1r confocal microscope using a 40 × 1.3 NA oil objective. Images were acquired using 3D Z-stacks through the entire DRG section. Images of DRG ATF3 protein expression were then converted to a maximum intensity projection image in order to visualize signal throughout the whole tissue section. Images of sciatic nerve ATF3/CD68 co-expression macrophages were single optical sections. Each collection of images was taken using the same exposure, gain, and LUTs settings. Quantification of macrophages used 8 regions of interest (140 μm × 140 μm) that were placed on the transmitted light image of the fasciculated axons of the sciatic nerve using the DIC view, and then, using the DAPI and FITC channels, individual CD68 positive cells were counted. The average number of cells per region was compared across conditions. 

### 5.14. Statistical Analysis

All statistical analyses were performed using GraphPad 9.5.1 Prism Software. Quantification of the data from the von Frey was done using Chaplan et al.’s method to calculate the 50% withdrawal thresholds [70] (50% withdrawal threshold = (10 [X_f_ + Kδ])/10,000)) to track of pain-like behavior and relief. For behavioral analysis, the values for each time point were then analyzed by two-way ANOVA to compare sex and condition. Tukey’s post hoc analysis for multiple comparisons of group means was performed following two-way ANOVA. The confidence interval was 95%, and the behavioral data displayed are presented as the mean ± SEM. 

The immunofluorescent imaging data comparing macrophage infiltration used a one-way ANOVA to treatment groups within each sex. This ANOVAs was secondarily evaluated using Tukey’s post hoc analysis for multiple comparisons. All immunofluorescent imaging data were calculated with a confidence interval of 95% and are displayed as the mean ± SEM with individual values plotted on the bar.

## Figures and Tables

**Table 1:** Top 25 significantly up-regulated genes in the female DRG following CCI injury of the sciatic nerve.

Gene ID	FDR	Fold Change	ENSMBL Gene ID
Npy	3.97 × 10^−42^	12.38	ENSRNOG00000046449
Atf3	2.17 × 10^−23^	7.83	ENSRNOG00000003745
Flrt3	2.46 × 10^−23^	2.51	ENSRNOG00000004874
Igfbp3	4.67 × 10^−15^	1.8	ENSRNOG00000061910
Gap43	8.54 × 10^−15^	1.58	ENSRNOG00000001528
Cacna2d1	7.36 × 10^−13^	1.91	ENSRNOG00000033531
Ktn1	1.04 × 10^−12^	1.53	ENSRNOG00000012255
Stac2	2.03 × 10^−12^	8.59	ENSRNOG00000004805
Gal	5.39 × 10^−12^	2.92	ENSRNOG00000015156
Gadd45a	7.04 × 10^−12^	2.25	ENSRNOG00000005615
Vip	9.71 × 10^−11^	18.83	ENSRNOG00000018808
AC130970.1	1.41 × 10^−10^	3.77	ENSRNOG00000042445
Csrp3	3.07 × 10^−10^	43.49	ENSRNOG00000014327
Ccl2	1.04 × 10^−9^	2.76	ENSRNOG00000007159
Sema6a	2.08 × 10^−9^	2.06	ENSRNOG00000004033
gene:ENSRNOG00000067276	2.18 × 10^−9^	1.81	ENSRNOG00000067276
Il7r	2.44 × 10^−9^	1.98	ENSRNOG00000065741
Prkar2b	3.04 × 10^−9^	1.46	ENSRNOG00000009079
Bdnf	8.06 × 10^−9^	2.14	ENSRNOG00000047466
Vgf	1.52 × 10^−8^	2.25	ENSRNOG00000001416
Stmn4	7.06 × 10^−8^	1.83	ENSRNOG00000053334
Sox11	1.17 × 10^−7^	2.53	ENSRNOG00000030034
Acsl4	1.23 × 10^−7^	1.43	ENSRNOG00000019180
Hs2st1	1.53 × 10^−7^	1.44	ENSRNOG00000012549
Il13ra1_2	1.69 × 10^−7^	1.76	ENSRNOG00000013170

**Table 2:** Top 25 significantly down-regulated genes in the female DRG following CCI injury of the sciatic nerve.

Gene ID	FDR	Fold Change	ENSMBL Gene ID
Sema7a	2.41 × 10^−21^	−1.82	ENSRNOG00000007687
Igfbp6	7.15 × 10^−15^	−2.12	ENSRNOG00000010977
RT1-CE16	8.18 × 10^−15^	−3.45	ENSRNOG00000065254
Tagln	4.07 × 10^−11^	−2.27	ENSRNOG00000017628
Iscu	9.71 × 10^−11^	−1.51	ENSRNOG00000000701
Hapln4	7.04 × 10^−10^	−1.9	ENSRNOG00000049949
Gsn	2.18 × 10^−9^	−1.46	ENSRNOG00000018991
Lgi3	2.08 × 10^−8^	−1.44	ENSRNOG00000011323
Hcn2	1.02 × 10^−7^	−1.46	ENSRNOG00000008831
gene:ENSRNOG00000070982	1.17 × 10^−7^	−2.08	ENSRNOG00000070982
Capg	1.17 × 10^−7^	−3.01	ENSRNOG00000013668
gene:ENSRNOG00000006471	1.51 × 10^−7^	−1.69	ENSRNOG00000006471
gene:ENSRNOG00000062930	1.97 × 10^−7^	−1.95	ENSRNOG00000062930
Fxyd6	2.21 × 10^−7^	−1.44	ENSRNOG00000016412
Islr	3.48 × 10^−7^	−1.76	ENSRNOG00000048924
Fxyd7	3.63 × 10^−7^	−1.4	ENSRNOG00000021067
Abhd8	3.96 × 10^−7^	−1.73	ENSRNOG00000000054
Tlcd3b	4.91 × 10^−7^	−1.4	ENSRNOG00000019914
Sptb	4.94 × 10^−7^	−1.39	ENSRNOG00000006911
Kcnh2	6.60 × 10^−7^	−1.42	ENSRNOG00000009872
Kcnc3	7.18 × 10^−7^	−1.41	ENSRNOG00000019959
Fbln2	7.35 × 10^−7^	−1.37	ENSRNOG00000007338
Kcns1	7.67 × 10^−7^	−1.67	ENSRNOG00000013681
Oaz1	1.08 × 10^−6^	−1.4	ENSRNOG00000019459
Bphl	1.08 × 10^−6^	−1.92	ENSRNOG00000017577

**Table 3:** Top 25 significantly up-regulated genes in the male DRG following CCI injury of the sciatic nerve.

Gene ID	FDR	Fold Change	ENSMBL Gene ID
Npy	2.67 × 10^−20^	5.79	ENSRNOG00000046449
Thbs1	9.51 × 10^−14^	1.88	ENSRNOG00000045829
Cfh	1.54 × 10^−9^	1.49	ENSRNOG00000030715
NEWGENE_1308171	1.77 × 10^−7^	1.58	ENSRNOG00000029792
RT1-Ba	5.72 × 10^−7^	1.94	ENSRNOG00000000451
Atf3	1.26 × 10^−6^	4.12	ENSRNOG00000003745
gene:ENSRNOG00000067832	1.55 × 10^−6^	41.86	ENSRNOG00000067832
Lmo7	1.59 × 10^−6^	1.62	ENSRNOG00000060775
Slc13a4	2.38 × 10^−6^	1.63	ENSRNOG00000011184
H3f3c	2.63 × 10^−6^	9.77	ENSRNOG00000032401
Phldb2	2.88 × 10^−6^	1.43	ENSRNOG00000002171
Fyb1	3.53 × 10^−6^	1.85	ENSRNOG00000013886
Aldh1a2	4.15 × 10^−6^	2.52	ENSRNOG00000055049
Cp	4.44 × 10^−6^	1.37	ENSRNOG00000011913
Sema3c	7.25 × 10^−6^	1.33	ENSRNOG00000006526
Fbln1	9.61 × 10^−6^	1.58	ENSRNOG00000014137
Emp1	1.65 × 10^−5^	1.55	ENSRNOG00000008676
Slc6a20	3.23 × 10^−5^	1.85	ENSRNOG00000068642
Thbd	3.55 × 10^−5^	1.55	ENSRNOG00000004687
Cilp	4.37 × 10^−5^	2.43	ENSRNOG00000029911
Crh	5.12 × 10^−5^	33.5	ENSRNOG00000012703
Slc6a13	5.12 × 10^−5^	1.99	ENSRNOG00000012876
Lcp1	5.12 × 10^−5^	1.3	ENSRNOG00000010319
Cfb	5.12 × 10^−5^	1.61	ENSRNOG00000051235
Fat4	6.83 × 10^−5^	1.32	ENSRNOG00000028335

**Table 4:** Top 25 significantly down-regulated genes in the male DRG following CCI injury of the sciatic nerve.

Gene ID	FDR	Fold Change	ENSMBL Gene ID
Egr2	1.47 × 10^−20^	−2.39	ENSRNOG00000000640
Zbtb16	1.27 × 10^−12^	−3.05	ENSRNOG00000029980
Myh2	1.27 × 10^−12^	−8.77	ENSRNOG00000065740
Sptbn5	1.39 × 10^−10^	−1.45	ENSRNOG00000059260
Tnnc2	6.32 × 10^−8^	−4.81	ENSRNOG00000015155
Dbp	7.66 × 10^−8^	−1.49	ENSRNOG00000021027
Ttn	1.48 × 10^−7^	−1.6	ENSRNOG00000069271
Tnnt3	1.77 × 10^−7^	−3.66	ENSRNOG00000020332
Mertk	1.90 × 10^−7^	−1.44	ENSRNOG00000017319
Tsc22d3	4.98 × 10^−7^	−1.38	ENSRNOG00000056135
Eno3	6.24 × 10^−7^	−2.42	ENSRNOG00000004078
Gpd1	6.88 × 10^−7^	−1.73	ENSRNOG00000056457
Sgk1	1.55 × 10^−6^	−1.58	ENSRNOG00000011815
AABR07005775.1	1.55 × 10^−6^	−3.71	ENSRNOG00000047124
Neb	4.44 × 10^−6^	−1.7	ENSRNOG00000006783
Hif3a	7.25 × 10^−6^	−5.79	ENSRNOG00000017198
Acta1	7.25 × 10^−6^	−3.23	ENSRNOG00000017786
Per1	8.28 × 10^−6^	−1.94	ENSRNOG00000007387
Aatk	1.14 × 10^−5^	−1.32	ENSRNOG00000004392
Fkbp5	1.19 × 10^−5^	−1.56	ENSRNOG00000022523
Mb	1.30 × 10^−5^	−5.5	ENSRNOG00000004583
Prx	1.34 × 10^−5^	−1.3	ENSRNOG00000018369
Mylpf	2.18 × 10^−5^	−3.55	ENSRNOG00000017645
Ntng2	3.12 × 10^−5^	−1.34	ENSRNOG00000013694
Atp1a3	3.38 × 10^−5^	−1.28	ENSRNOG00000020263

**Table 5:** The significantly up-regulated genes in the female DRG following reduction of neuroinflammation with COX-2 inhibiting theranostic nanoemulsion.

Gene ID	FDR	Fold Change	ENSMBL Gene ID
Myom3	1.10 × 10^−4^	9.43	ENSRNOG00000032994
Grhl3	8.06 × 10^−4^	13.29	ENSRNOG00000029427
Txnip	2.92 × 10^−3^	1.35	ENSRNOG00000021201
Hba-a3	4.00 × 10^−2^	2.51	ENSRNOG00000047321
Sptb	0.02	1.31	ENSRNOG00000006911
Gas2l3	0.03	1.3	ENSRNOG00000063755

**Table 6:** The significantly down-regulated genes in the female DRG following reduction of neuroinflammation with COX-2 inhibiting theranostic nanoemulsion.

Gene ID	FDR	Fold Change	ENSMBL Gene ID
Ass1	3.44 × 10^−4^	−2.4	ENSRNOG00000008837
Vgf	4.22 × 10^−4^	−1.63	ENSRNOG00000001416
Atf3	8.06 × 10^−4^	−2.09	ENSRNOG00000003745
Sox11	1.35 × 10^−3^	−1.66	ENSRNOG00000030034
Lce1f_2	8.47 × 10^−3^	−5.85	ENSRNOG00000066187
Sema6a	1.00 × 10^−2^	−1.67	ENSRNOG00000004033
AABR07007068.1	2.00 × 10^−2^	−1.3	ENSRNOG00000017438
Gadd45a	3.00 × 10^−2^	−1.49	ENSRNOG00000005615
Jun	4.00 × 10^−2^	−1.28	ENSRNOG00000026293
Rbp4	5.00 × 10^−2^	−1.68	ENSRNOG00000015518

**Table 7:** Top 25 most significantly up-regulated genes in the male DRG following reduction of neuroinflammation with COX-2 inhibiting theranostic nanoemulsion.

Gene ID	FDR	Fold Change	ENSMBL Gene ID
S100a9	9.37 × 10^−30^	5.68	ENSRNOG00000011483
S100a8	4.11 × 10^−28^	6.24	ENSRNOG00000011557
Mmp8	2.84 × 10^−20^	7.53	ENSRNOG00000009907
Ngp	1.18 × 10^−18^	5.2	ENSRNOG00000024330
Ighm	7.44 × 10^−17^	3.2	ENSRNOG00000034190
Ccl21	3.78 × 10^−16^	4.15	ENSRNOG00000034290
Slc4a1	2.75 × 10^−13^	3.69	ENSRNOG00000020951
Fcnb	8.83 × 10^−13^	5.16	ENSRNOG00000009342
Ccn3	1.45 × 10^−12^	2.77	ENSRNOG00000008697
Mfap4	1.60 × 10^−12^	2.41	ENSRNOG00000045683
NEWGENE_1308171	3.27 × 10^−11^	1.45	ENSRNOG00000029792
Mpo	2.04 × 10^−10^	5.12	ENSRNOG00000008310
Ptprc	2.46 × 10^−10^	1.81	ENSRNOG00000000655
AABR07060872.1	4.76 × 10^−10^	4.5	ENSRNOG00000049829
Serpinb1b	5.34 × 10^−10^	11.78	ENSRNOG00000034229
Mki67	5.34 × 10^−10^	2.22	ENSRNOG00000028137
Ccn2	1.13 × 10^−9^	2.03	ENSRNOG00000015036
Sell	1.25 × 10^−9^	3.82	ENSRNOG00000002776
Lyz2	1.25 × 10^−9^	1.67	ENSRNOG00000005825
Prg2	3.12 × 10^−9^	4.31	ENSRNOG00000008394
RatNP-3b	4.14 × 10^−9^	7.22	ENSRNOG00000038135
Hemgn	1.29 × 10^−8^	3.6	ENSRNOG00000009436
Slfn4_1	1.39 × 10^−8^	2.24	ENSRNOG00000057092
Plac8	6.24 × 10^−8^	3.18	ENSRNOG00000002217
Acta2	1.26 × 10^−7^	1.77	ENSRNOG00000058039

**Table 8:** Top 25 most significantly down-regulated genes in the male DRG reduction of neuroinflammation with COX-2 inhibiting theranostic nanoemulsion.

Gene ID	FDR	Fold Change	ENSMBL Gene ID
Dbp	8.42 × 10^−15^	−1.72	ENSRNOG00000021027
Prim1	3.56 × 10^−5^	−2.01	ENSRNOG00000031993
Hmgb1	6.42 × 10^−5^	−2.03	ENSRNOG00000058908
Per1	9.79 × 10^−5^	−1.53	ENSRNOG00000007387
Zbtb16	2.66 × 10^−4^	−1.89	ENSRNOG00000029980
Gjc3	5.34 × 10^−4^	−1.29	ENSRNOG00000001329
Sgk1	6.90 × 10^−4^	−1.3	ENSRNOG00000011815
Clvs1	1.03 × 10^−3^	−2.39	ENSRNOG00000006919
Chrna3	1.92 × 10^−3^	−1.38	ENSRNOG00000013829
Acer2	1.95 × 10^−3^	−1.52	ENSRNOG00000007637
Nptx1	2.13 × 10^−3^	−1.23	ENSRNOG00000003741
Crh	3.52 × 10^−3^	−21.61	ENSRNOG00000012703
Trim45	4.35 × 10^−3^	−2.29	ENSRNOG00000015347
AABR07034445.1	7.52 × 10^−3^	−2.27	ENSRNOG00000038610
Ntng1	7.98 × 10^−3^	−1.25	ENSRNOG00000031136
Klf9	8.75 × 10^−3^	−1.23	ENSRNOG00000014215
Sema7a	8.99 × 10^−3^	−1.23	ENSRNOG00000007687
RGD1564899	0.01	−2.2	ENSRNOG00000049942
Nr1d1	0.02	−2.2	ENSRNOG00000009329
Gnai1	0.02	−1.21	ENSRNOG00000057096
Kcng1	0.03	−1.39	ENSRNOG00000054314
Chst2	0.03	−1.19	ENSRNOG00000047734
Slitrk2	0.03	−1.19	ENSRNOG00000011562
Svip	0.03	−1.19	ENSRNOG00000037137
Ncam2	0.03	−1.19	ENSRNOG00000002126

**Table 9:** List of differentially expressed genes that show unique patterns of differential RNA expression across sex and or treatment. Table information adapted from output data provided by Metascape.com.

Gene Name	Full Gene Name	Gene Ontology	Protein Function	Pattern of Expression
**Atf3**	Activating transcription factor 3	GO:0061394 regulation of transcription from RNA polymerase II promoter in response to arsenic-containing substance; GO:1903984 positive regulation of TRAIL-activated apoptotic signaling pathway; GO:1903121 regulation of TRAIL-activated apoptotic signaling pathway	Transcription factors: Basic domains; Predicted intracellular proteins	Up-regulated in both sexes following CCI, down-regulated with relief in females
Sema6a	Semaphorin 6A	GO:0106089 negative regulation of cell adhesion involved in sprouting angiogenesis; GO:0106088 regulation of cell adhesion involved in sprouting angiogenesis; GO:1900747 negative regulation of vascular endothelial growth factor signaling pathway	Predicted intracellular proteins	Up-regulated in both sexes following CCI, down-regulated with relief in females
Vgf	VGF nerve growth factor inducible	GO:0043084 penile erection; GO:0007620 copulation; GO:0002021 response to dietary excess	Predicted secreted proteins	Up-regulated in both sexes following CCI, down-regulated with relief in females
Flrt3	Fibronectin leucine rich transmembrane protein 3	GO:0003345 proepicardium cell migration involved in pericardium morphogenesis; GO:0003344 pericardium morphogenesis; GO:0060973 cell migration involved in heart development	Disease related genes; Human disease related genes: Endocrine and metabolic diseases: Hypothalamus and pituitary gland diseases	Up-regulated in both sexes following CCI. No change with relief.
**Crh**	Corticotropin releasing hormone	GO:0042322 negative regulation of circadian sleep/wake cycle, REM sleep; GO:0010841 positive regulation of circadian sleep/wake cycle, wakefulness; GO:0042321 negative regulation of circadian sleep/wake cycle, sleep	Predicted secreted proteins	Up-regulated in both sexes following CCI, down-regulated with relief in males
Prim1	DNA primase subunit 1	GO:0006269 DNA replication, synthesis of RNA primer; GO:0006270 DNA replication initiation; GO:0006261 DNA-templated DNA replication	Predicted intracellular proteins	Up-regulated in males following CCI, down-regulated with relief in males
Prx	Periaxin	GO:0032287 peripheral nervous system myelin maintenance; GO:0043217 myelin maintenance; GO:0022011 myelination in peripheral nervous system	Disease related genes; Human disease related genes: Nervous system diseases: Other nervous and sensory system diseases; Human disease related genes: Nervous system diseases: Neurodegenerative diseases; Predicted intracellular proteins	Down-regulated in both sexes following CCI, no significant change with treatment
Myh2	Myosin Heavy Chain 2	GO:0030049 muscle filament sliding; GO:0033275 actin-myosin filament sliding; GO:0070252 actin-mediated cell contraction	Disease related genes; Human disease related genes: Musculoskeletal diseases: Muscular diseases; Predicted intracellular proteins	Up-regulated in females following CCI, down-regulated in males following CCI
Tnnt3	Troponin T3	GO:1903612 positive regulation of calcium-dependent ATPase activity; GO:1903610 regulation of calcium-dependent ATPase activity; GO:0003009 skeletal muscle contraction	Disease related genes; Predicted intracellular proteins; Human disease related genes: Congenital malformations: Other congenital malformations	Up-regulated in females following CCI, down-regulated in males following CCI

## Data Availability

Data is contained within the article or Supplementary Materials.

## Supplementary Materials

The following supporting information can be downloaded at: https://www.mdpi.com/article/10.3390/ijms24119163/s1.
