# Supplementary material for: RNA-Seq Reveals Sex Differences in Gene Expression during Peripheral Neuropathic Inflammation and in Pain Relief from a COX-2 Inhibiting Theranostic Nanoemulsion
Source: Int J Mol Sci. 2023 May 23;24(11):9163. doi: 10.3390/ijms24119163 (PMC10252608; doi:10.3390/ijms24119163)
Supplement: Supplementary file 1 [file ijms-24-09163-s001.zip › ijms-2342223-supplementary.pdf]

International Journal of *Molecular Sciences*

MDPI

Supplementary Data

RNA-seq reveals sex differences in gene expression during peripheral neuropathic inflammation and in pain relief from a COX-2 inhibiting theranostic nanoemulsion

Brooke Deal [1,2], Katherine Phillips [1,2], Caitlin Crelli [2,3], Jelena M. Janjic [2,3,*] and John A. Pollock [1,2,*]

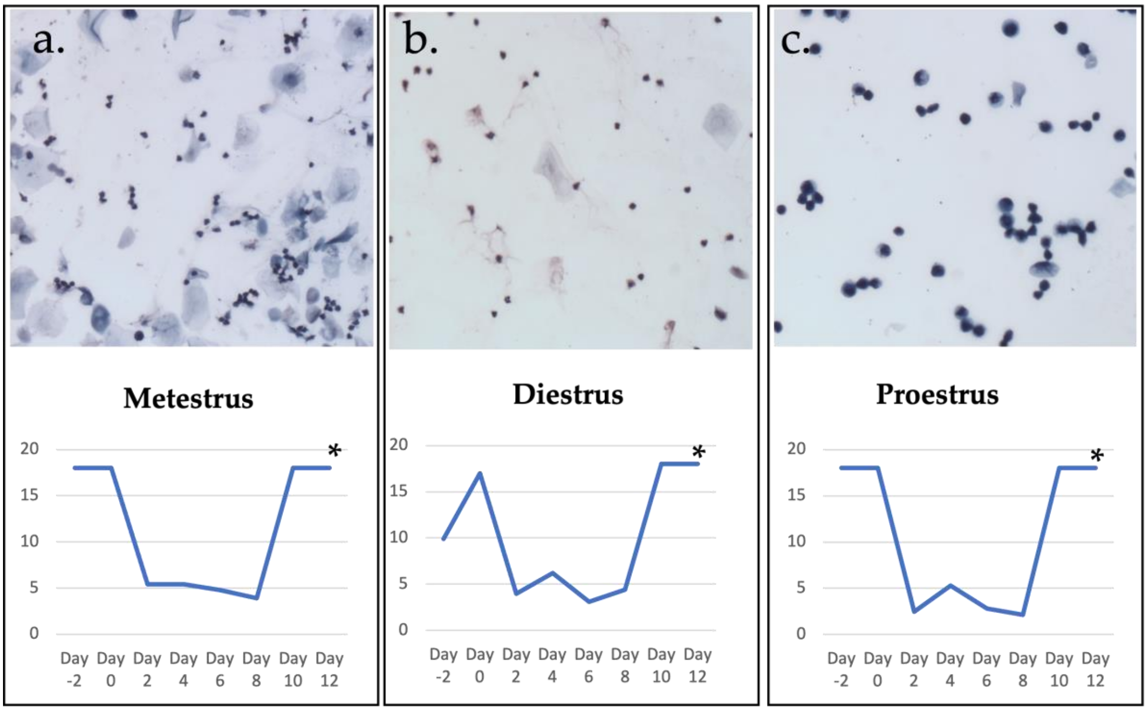

Supplementary Figure 1. Vaginal smears reveal the stage of the estrus cycle on Day 12 (*) for three different CXB-NE treated females presented with their individual behavioral profiles. (a) 10X CXB-NE female 1 was in metestrus on day 12, which is distinguished by neutrophils intermixed between epithelial cells. Additionally, 10X CXB-NE female 1 fully recovers to their presurgical baseline withdrawal threshold. (b) 10X CXB-NE female 2 was in diestrus on day 12, which can be characterized by the majority of cells present being leukocytes.10X CXB-NE female 2 fully recovers to their presurgical baseline with treatment. (c) 10X CXB-NE female 3 is in proestrus on day 12, which is characterized by the majority cells being nucleated epithelial cells. 10X CXB-NE female #3 also fully recovers to presurgical baseline.

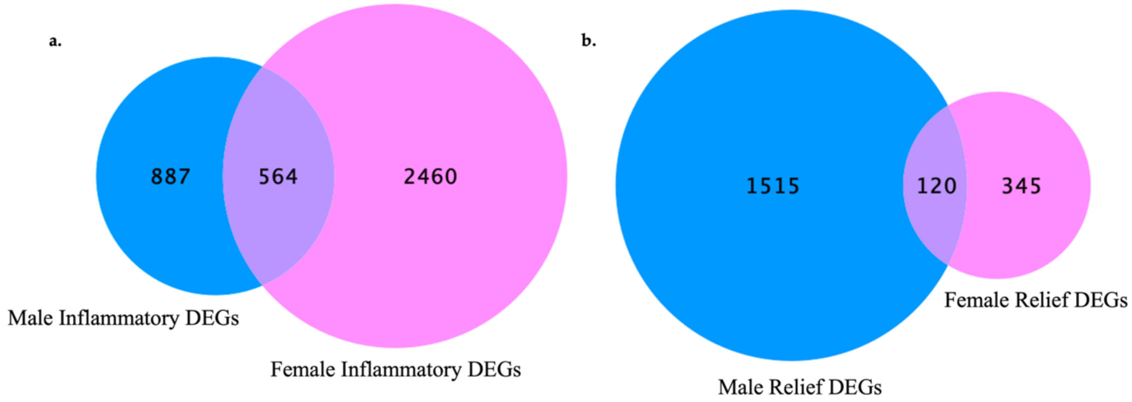

Supplementary Figure 2. Venn Diagram of differentially expressed genes using p-value ≤ 0.05. (a) Male and female differentially expressed genes resulting from induction of neuroinflammation. **(b)** Male and female differentially expressed genes in response to celecoxib theranostic nanoemulsion treatment.

Supplementary Table 1. List of 90 genes from the male heat map gene tree (Figure 3a). Genes are listed in order of the gene heatmap tree from left to right and then top to bottom.

Scd2	Mbp	Mpz
Pmp22	Fth1	Tuba1b
Actb	gene:ENSRNOG00000051885	Tubb3
Cd74	Prph	Ctsb
Actg1	Stmn2	Npy
Anxa2	Sparc	Ubb
gene:ENSRNOG00000047931	Gapdh	Serpinb1b
Col3a1	Col1a1	Col1a2
Igfbp5	Hba-a2	Hbb-b1
gene:ENSRNOG00000062930	Mt-nd1	Mt-nd4
Mt-nd5	Mt-nd2	Mt-cyb
Mt-nd3	Cst3	Mt-co1
Mt-atp6	Mt-co3	NEWGENE_1308171
Ptgds	Ahnak	Eef1a1
Mt-co2	Mt-atp8	Apod
Dcn	Hspa8	Ywhaz
gene:ENSRNOG00000065195	Sptan1	Sptbn1
Prune2	Hsp90aa1	Fstl1
Rtn3	Snap25	Nefh
Nefm	Nefl	Ywhah
Atp1b1	Dync1h1	Kif1a
Map1a	Atp1a1	Epb41l3
Map1b	Rtn4	Kif5a
Kcna1	Ywhag	Pgd
Calm1_2	Cltc	Hsp90ab1
Dst	Ank2	Gnas_2
Ttn	Neb	Acta1
Myh4	AABR07005775.1	Apoe
Uchl1	Zwint	Sncg
Thy1	Scn7a	Rgs4

Supplementary Table 2. List of 90 genes from the female heat map gene tree (Figure 3b). Genes are listed in order of the gene heatmap tree from left to right and then top to bottom.

Ttn	Myh4	Dpysl2
Kif5a	gene:ENSRNOG00000062930	Scd2
Map1b	Dync1h1	Atp1a1
Nefm	Map1a	Ywhag
Atp1a3	Atp11a	Kcna1
Nefh	Nefl	Ndrg4
Vamp1	Fth1	Mbp
Mpz	Pmp22	Cst3
Apoe	Uchl1	Rtn3
Sptan1	Fstl1	Mt-nd3
Nedd4	Hsp90aa1	Eef1a1
Hspa8	Mt-co1	Mt-co2
Tmem176b	gene:ENSRNOG00000047931	Anxa2
Sncg	Gapdh	Thy1
Rgs4	Serpinb1b	Mx2
Dst	Epb41l3	Ank2
Sptbn1	Tuba1b	Tubb3
Prph	Stmn2	Npy
Gap43	Cacna2d1	Spp1
Cd59	Ywhaz	Akap6
Pafah1b1	Calm1_2	Cltc
Usp9x	Scn9a	Prune2
Mt-atp8	Scn7a	Apod
Hspa5	Actg1	Hsp90ab1
Ahnak	Dcn	Col1a1
Col1a2	Igfbp5	Col3a1
Mt-nd6	Hba-a2	Hbb-b1
Actb	Mt-cyb	Mt-atp6
Mt-nd2	Mt-co3	Mt-nd4l
Mt-nd5	Mt-nd4	Mt-nd1

Supplementary Table 3. List of forward and reverse primers used in qPCR validation.

Gene	Forward Primer	Reverse Primer
GAPDH	5′-GGCACAGTCAAGGCTGAGAATG-3′	5′-ATGGTGGTGAAGACGCCAGTA-3′
ATF3	5′-CCTGCAGAAGGAGTCAGAGAA-3′	5′-CGTTCTGAGCCCGGACGATA-3′
Sema6a	5′-AGCAAGACATAGAGCGTGGC-3′	5′-TGGCACGCCCATTCAGT-3′

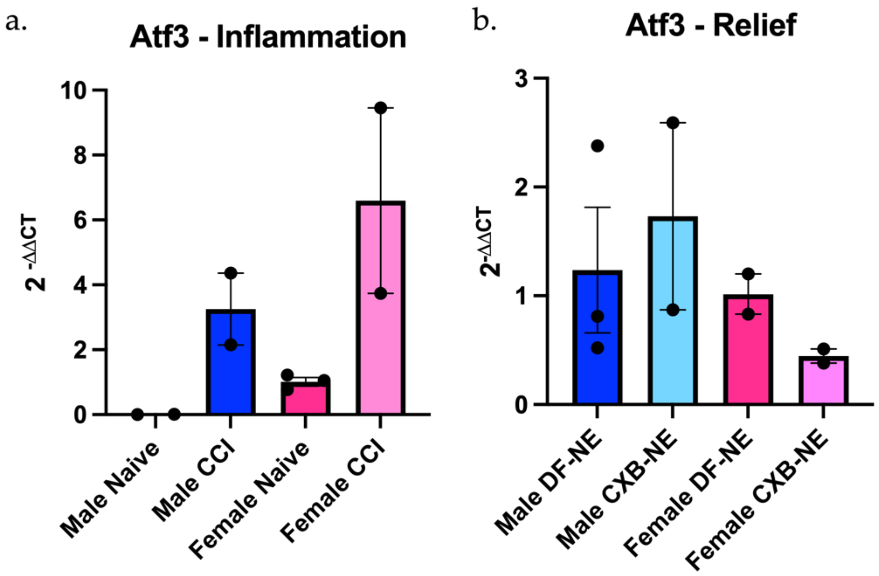

Supplementary Figure 3. Analysis of qPCR reveal changes in RNA expression for *ATF3* (a) Both male and female CCI DRGs exhibit an increase in RNA expression for *ATF3* following CCI neuronal injury and neuroinflammation (male naïve n = 2, male CCI n = 2, female naïve n = 3, female CCI n = 2). **(b)** Treatment with 10X CXB-NE results in a decrease in *ATF3* RNA expression in females in contrast to their DF-NE counterparts. Male *ATF3* RNA expression appears to be unaffected by the celecoxib theranostic nanoemulsion treatment (male DF-NE n = 3, male 10X CXB-NE n = 2, female DF-NE n = 2, female 10X CXB-NE n = 2).

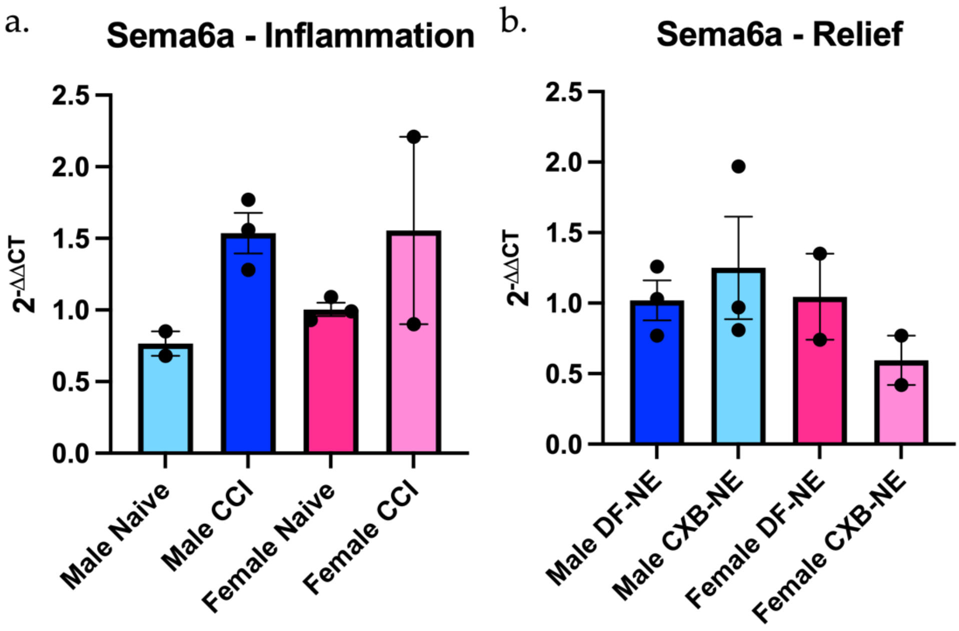

Supplementary Figure 4. Analysis of qPCR reveals changes in RNA expression for *SEMA6A*. (a) Both male and female CCI DRGs exhibit an increase in RNA expression of *SEMA6A* following CCI neuronal injury and neuroinflammation (male naïve n = 2, male CCI n = 3, female naïve n = 3, female CCI n = 2). **(b)** Treatment with 10X CXB-NE results in a decrease in *SEMA6A* RNA expression in females in contrast to their DF-NE counterparts. Male *SEMA6A* RNA expression appears to be unaffected by the celecoxib theranostic nanoemulsion treatment (male DF-NE n = 3, male 10X CXB-NE n = 3, female DF-NE n = 2, female 10X CXB-NE n = 2).

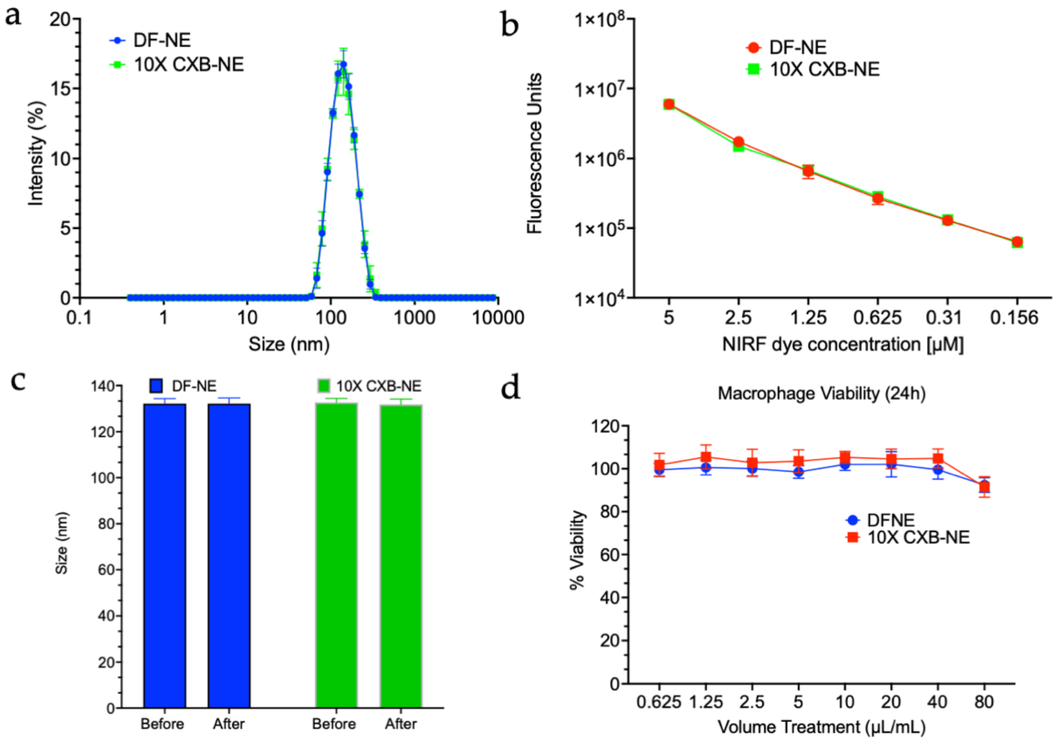

Supplementary Figure 5. In vitro characterization of 10X CXB-NE and DF-NE theranostic nanoemulsions used in this study. (a) The two nanoemulsions show identical size distribution profile as measured by dynamic light scattering and average size ~130nm. **(b)** Nanoemulsions show equivalent fluorescence signal profiles as measured by NIRF imaging on LiCOR Odyssey at the same optimized conditions. **(c)** Nanoemulsions do not change droplet size upon filtration through 0.22 μm filter. (**d)** Nanoemulsions do not reduce cell viability when tested in RAW 264.7 cells in vitro using Cell Titer Glo luminescence assay. Data represents the average ± CD (n=6/condition). Data is processed on Graph Pad Prism 9. Methods for nanoemulsions characterizations used here have been reported previously [1-3].

1. Herneisey, M. and J.M. Janjic, *Multiple Linear Regression Predictive Modeling of Colloidal and Fluorescence Stability of Theranostic Perfluorocarbon Nanoemulsions.* Pharmaceutics, 2023. **15**(4).
2. Herneisey, M., et al., *Development of Theranostic Perfluorocarbon Nanoemulsions as a Model Non-Opioid Pain Nanomedicine Using a Quality by Design (QbD) Approach.* AAPS PharmSciTech, 2019. **20**(2): p. 65.
3. Nichols, J.M., et al., *Tracking macrophages in diabetic neuropathy with two-color nanoemulsions for near-infrared fluorescent imaging and microscopy.* J Neuroinflammation, 2021. **18**(1): p. 299.
